# Anionic Boron-Cluster BODIPY Conjugates As Promising Photosensitizers for Targeted Antimicrobial Photodynamic Therapy

**DOI:** 10.1021/acsomega.5c07634

**Published:** 2025-09-16

**Authors:** Javier Ordóñez-Hernández, Andromeda-Celeste Gómez, Jordi Hernando, Marina Quevedo, Juan Camilo Ortíz, Daniel Yero, Isidre Gibert, Rosario Núñez

**Affiliations:** † Inorganic Materials and Catalysis Laboratory (LMI), Institut de Ciència de Materials de Barcelona (ICMAB-CSIC), Campus de la UAB, Bellaterra (Cerdanyola del Vallès), Barcelona 08193, Spain; ‡ Institut de Biotecnologia i de Biomedicina, 98700 Universitat Autònoma de Barcelona (UAB), Bellaterra (Cerdanyola del Vallès), Barcelona 08193, Spain; § Departament de Genètica i de Microbiologia, Universitat Autònoma de Barcelona (UAB), Bellaterra (Cerdanyola del Vallès), Barcelona 08193, Spain; ∥ Departament de Química, Universitat Autònoma de Barcelona (UAB), Bellaterra (Cerdanyola del Vallès), Barcelona 08193, Spain

## Abstract

We report the design, synthesis, photophysical characterization, and antimicrobial evaluation of three novel anionic boron-cluster–BODIPY conjugates, **BDP-FES** (BODIPY-ferrabisdicarbollide), **BDP-COS** (BODIPY-cobaltabisdicarbollide), and **BDP-B**_**12**_ (BODIPY-*closo*-dodecaborate), as photosensitizers for antimicrobial photodynamic therapy (aPDT). Covalent attachment of anionic boron clusters to the iodinated BODIPY scaffold was confirmed by multinuclear NMR spectroscopy and resulted in red-shifted absorption (λ_abs,max_ = 545 nm) and emission (λ_f,max_ = 600–611 nm) maxima, along with pronounced suppression of fluorescence quantum yields due to heavy-atom-promoted triplet state generation. While singlet oxygen generation decreased compared to the parent **BDP-I**_**2**_, the new conjugates, particularly **BDP-B**_**12**_, demonstrated potent, light-dependent bactericidal activity against multidrug-resistant Gram-positive bacteria, including *Staphylococcus aureus*, *Enterococcus faecium*, and *Enterococcus raffinosus*, with complete eradication of *S. aureus* at 5 μM under green light irradiation. All conjugates exhibited negligible toxicity in the dark, indicating selective photodynamic activity and an improved safety profile. These findings establish anionic boron clusters as versatile molecular tools to modulate the photophysical and biological properties of BODIPY-based photosensitizers. Our results lay the groundwork for the development of next-generation, biocompatible photodynamic agents for targeted disinfection and infection control, bridging inorganic chemistry and biomedical applications.

## Graphical abstract

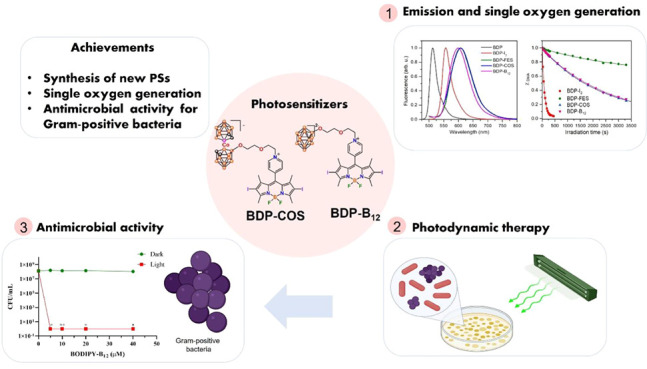

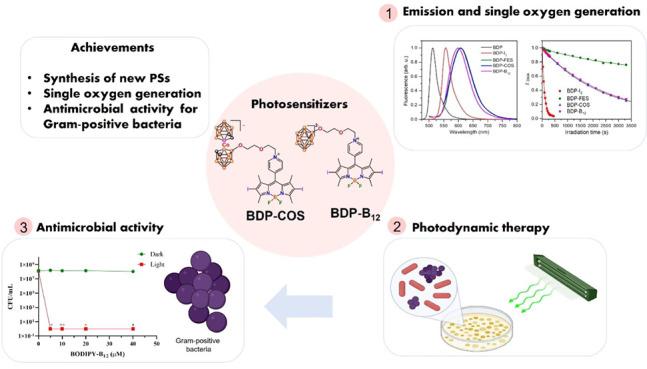

## 1 Introduction

The growing resistance of microorganisms (bacteria, viruses, and fungi) to antimicrobials and antiseptics represents one of the most pressing challenges in modern medicine and biology.^1^ According to the European Center for Disease Prevention and Control (ECDC), antimicrobial resistance (AMR) poses a critical global health threat, with multidrug-resistant pathogens causing over 35000 annual deaths in the EU alone and projected to claim 10 million lives yearly by 2050.^2,3^ Healthcare-associated infections, predominantly caused by multidrug-resistant (MDR) bacteria, continue to pose a significant public health threat. This underscores the urgent need to develop effective alternative strategies that operate via entirely different mechanisms. This crisis demands innovative therapeutic strategies beyond conventional antibiotics. Antimicrobial photodynamic therapy (aPDT) has emerged as a promising alternative, leveraging photosensitizers (PSs), light, and oxygen to generate cytotoxic reactive oxygen species (ROS) that eradicate pathogens via a multitarget mechanism, minimizing resistance development.

Antimicrobial photodynamic therapy (aPDT) has emerged as a promising strategy for surface disinfection in healthcare settings, particularly for medical equipment, high-touch surfaces, and implantable devices.^4,5^ This approach combines PSs, light, and molecular oxygen to generate toxic ROS, which induce oxidative damage in microbial cells.^6^ Unlike traditional antimicrobials, aPDT’s multitarget mechanism minimizes resistance development, making it effective against bacteria, fungi, viruses, and parasites, including drug-resistant strains.^7^

BODIPY fluorophores have arisen as versatile PSs in aPDT, offering a promising solution to combat drug-resistant pathogens.^8^ BODIPYs have a rich chemistry allowing substitution at various positions on the pyrrole rings and the boron center.^9,10^ This structural versatility allows fine-tuning of their spectroscopic and chemical properties.^11^ Due to their excellent photostability and fluorescence efficiency, BODIPYs are widely used as fluorescent probes and markers in biological research, including DNA sequencing, labeling, and imaging.^12–14^ In addition, they serve as fluorescent tags in synthetic chemistry and have applications in bioimaging, sensing, and therapeutic fields, including aza-BODIPYs, which extend the absorption/emission into the near-infrared region.^15^ Their versatile chemical structures, exceptional photophysical characteristics, high photostability, and efficient ROS generation make BODIPY derivatives highly effective against a broad spectrum of pathogens, including bacteria, fungi, and viruses.^8,16^ Recent studies have also demonstrated that BODIPY derivatives have potent biological activities such as antioxidant, antimicrobial, antibiofilm, and DNA cleavage capabilities, highlighting their potential in photodynamic therapy,^17–20^ and biomedical applications.^13^ Despite these advantages, classical BODIPY PSs face limitations in clinical translation, including poor aqueous solubility, ambient-light sensitivity reducing photoselectivity and limited biofilm penetration.

Anionic boron clusters (including cobaltabis­(dicarbollide) ([3,3′-Co­(1,2-C_2_B_9_H_11_)_2_]^−^, *o*-COSAN), ironbis­(dicarbollide) ([3,3′-Fe­(1,2-C_2_B_9_H_11_)_2_]^−^, *o*-FESAN), and *closo*-dodecaborate ([B_12_H_12_]^2–^) represent a versatile class of inorganic compounds with unique structural, physicochemical and functional properties.^21^ They demonstrate exceptional stability and highly tunable chemical properties, positioning them as outstanding candidates for a wide range of biomedical applications.^22–28^ Furthermore, recent studies have highlighted their notable antimicrobial activity, further broadening their potential utility.^29–32^ *o-*COSAN is characterized by low nucleophilicity and charge density,^33,34^ and exceptional stability.^35–37^ Its inherent hydrophobic nature imparts strong amphiphilic behavior to both its protonated and sodium salts,^38^ enhancing their solubility in both water-based and organic solvents.^39,40^ This amphiphilicity also drives their spontaneous assembly into micelles and monolayer vesicles in aqueous solutions.^41–43^ Due to these distinctive physicochemical traits, COSAN is able to cross lipid bilayers and accumulate within various types of living cells.^44–49^ Its superchaotropic nature enables dynamic association with lipid membranes, facilitating transport of hydrophilic cargo (e.g., peptides, drugs) across biological barriers.^50,51^ As far as we know, the biomedical research and applications of the paramagnetic *o*-FESAN have been significantly less investigated than its homologous *o*-COSAN.^52,53^ On the other hand, the dianion ([B_12_H_12_]^2–^) exhibits three-dimensional aromaticity and exceptional thermal/chemical stability.^54,55^ Functionalization via halogenation (e.g., B_12_X_12_^2–^, X = Cl, Br, I) enhances its superchaotropic character, enabling membrane transport of diverse cargo.^56,57^ Additionally, other anionic boron clusters as *nido*-*o*-carborane and its derivatives also display good bioactivity and photophysical properties.^58,59^

This study pioneers the integration of BODIPY cores with anionic boron clusters for aPDT. While neutral and anionic boron cluster-BODIPY conjugates have been explored for anticancer therapy and bioimaging,^60–64^ no prior work exists on their application in antimicrobial therapies. On the other hand, it is expected that the exceptional cellular uptake of boron cluster-BODIPY conjugates^46^ could enhance their activity against different bacterial pathogens. To validate this hypothesis, we have designed, synthesize and characterized three novel conjugates, **BDP-COS** (BODIPY-cobaltabisdicarbollide), **BDP-FES** (BODIPY-ferrabisdicarbollide), and **BDP-B**_**12**_ (BODIPY-*closo*-dodecaborate), which were tested as potential photosensitizers for antimicrobial photodynamic therapy. The research aimed to explore how the incorporation of anionic boron clusters modulates the photophysical properties of BODIPY, such as absorption and emission shifts, as well as singlet oxygen generation. Additionally, the study evaluated the light-dependent antimicrobial and bactericidal activity of these conjugates against Gram-positive and Gram-negative bacteria, while assessing their selectivity and solubility improvements over the parent compound. This work establishes anionic boron clusters as effective modifiers to enhance targeted aPDT against resistant Gram-positive pathogens. Our work establishes the first framework for boron-cluster-BODIPY hybrids in combating resistant pathogens throughout PDT, making these promising candidates for surface disinfection in healthcare settings and medical surfaces. This approach bridges inorganic boron chemistry and photodynamic microbiology, offering a drawing for next-generation antimicrobials.

## 2 Results and Discussion

### 2.1 Synthesis and Structural Characterization

The family of three new photosensitizers based on anionic boron-cluster-BODIPY conjugates, which include ferrabisdicarbollide (**BDP-FES**), cobaltabisdicarbollide (**BDP-COS**), and *closo*-dodecaborate (**BDP-B**_**12**_), was synthesized according to the synthetic methodology shown in Scheme 1. To synthesize the target compounds, the first step was the three-step preparation of the BODIPY core. First, a condensation reaction was conducted between 4-formylpyridine and 2,4-dimethylpyrrole, utilizing trifluoroacetic acid as a catalyst. This reaction yielded the corresponding dipyrromethane, which was oxidized using 2,3-dichloro-5,6-dicyano-1,4-benzoquinone (DDQ); finally, a complexation reaction with BF_3_·OEt_2_ and Et_3_N produced **BDP**, yielding 23% overall yield across the three steps. Afterward, an electrophilic substitution reaction on **BDP** was performed with an excess of iodine (I_2_) and iodic acid (IHO_3_) to obtain **BDP-I**_**2,**_ with 50% yield. Next, **BDP-I**_**2**_ reacted with the corresponding oxonium boron clusters *closo*-dodecaborate-dioxane,^65^ COSAN-dioxane,^66^ and FESAN-dioxane,^67^ which were prepared using methods reported in the literature. These reactions occur through a nucleophilic attack by the nitrogen atom of the pyridine moiety on the oxonium ring (Scheme 1). The PSs **BDP-FES** and **BDP-COS** were obtained with moderate yields of 59% and 64%, respectively. In contrast, **BDP-B**_**12**_ was isolated with a notably low yield (15%), attributed to the steric hindrance from the bulky [NBu_4_]^+^ cation in the oxonium-*closo*-dodecaborate cluster, which hampered the nucleophilic substitution reaction.

**Scheme 1:**
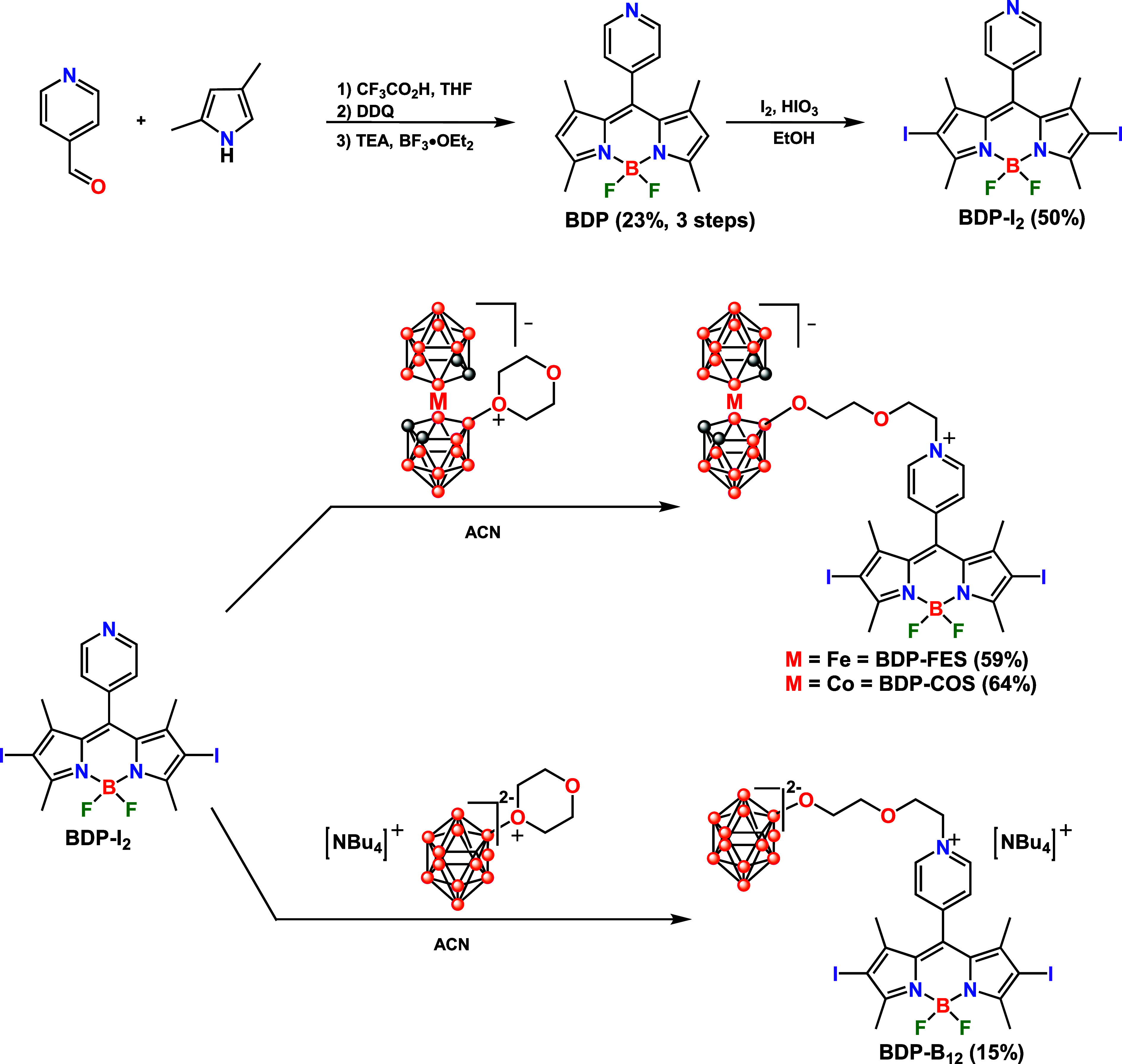
Synthetic Methodology Followed to Synthesize the PSs

All compounds were characterized by IR-ATR, ^1^H, ^1^H­{^11^B}, ^13^C and ^11^B­{^1^H} NMR spectroscopies (Figures S1–S18), absorption and emission spectroscopy, and elemental analysis. The IR-ATR spectra for all PSs based on boron-cluster BODIPY exhibit strong ν­(B–H) bands between 2520 and 2536 cm^–1^, confirming the presence of boron clusters. Additionally, the B–F bands are noticed at about 1521 cm^–1^ and 1530 cm^–1^, respectively, confirming the presence of the BODIPY core.

The ^1^H NMR spectra (Figures 1, S11, and S15) show two doublets at 9.63 and 8.64 ppm for **BDP-COS** and 9.96 and 8.40 ppm for **BDP-B**_**12**_, which correspond to the protons of the pyridyl ring bonded to the *meso* position of the BODIPY core. Additionally, two singlets at 2.68 and 1.61 ppm for **BDP-COS**, and at 2.63 and 1.57 ppm for **BDP-B**_**12**_, attributed to the protons of the methyl groups of the BODIPY core, are also observed. Furthermore, **BDP-COS** exhibits four resonances at 5.20, 4.29, 4.11, and 4.03 ppm, which are associated with the CH_2_ hydrogens of the ethylene glycol chain. These resonances appear at 5.21, 4.12, 3.74, and 3.67 ppm for the **BDP-B**_**12**_. The **BDP-COS** spectrum also exhibits a multiplet in the range of 3.75–3.70 ppm, which integrates for four protons corresponding to the C_cluster_–H (C_c_–H) of the cluster. In the case of **BDP-B**_**12**_, three multiplets at 3.48–3.42 ppm, 1.89–1.78 ppm, and 1.51–1.38 ppm, along with a triplet at 0.99 ppm, have been associated with cation [NBu_4_]^+^. On the other hand, a very different ^1^H NMR spectrum was observed for **BDP-FES** that, as expected, exhibits appreciable paramagnetic shifts of resonances in the range from δ −5.3 to +74 ppm, especially in those protons closer to the Fe­(III) center (Figure S7).^67^

**Figure 1:**
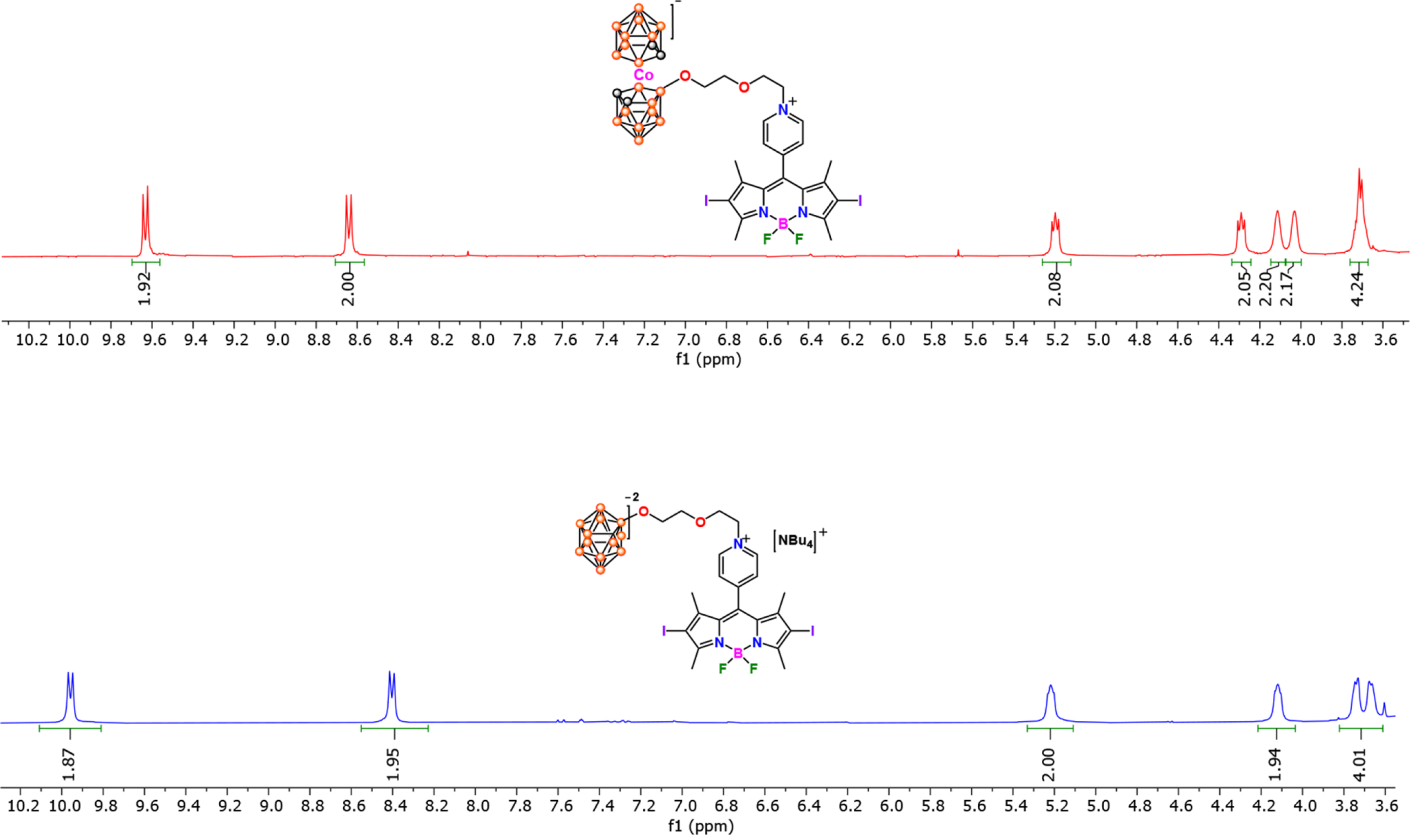
Partial view of the ^1^H NMR spectra in CO­(CD_3_)_2_ at 300 MHz. **BDP-COS** (up) and **BDP-B**_**12**_ (down).

The ^13^C NMR spectra of all PSs (Figures S8, S12, and S16) show resonances that correspond to the different carbons of the BODIPY core, the ethylene glycol chain, the cluster (FESAN and COSAN), and the [NBu_4_]^+^ cation in the case of **BDP-B**_**12**_. In the ^19^F NMR spectra (Figures S9, S13 and S17), a quadruple signal corresponding to the two fluorine atoms of the BODIPY core is observed at approximately −145 ppm with a *J*_B–F_ coupling constant between 31 and 32 Hz for all compounds. The ^11^B­{^1^H} NMR spectra confirmed the presence of the respective boron cluster: **BDP-FES** shows patterns in the range of 116 to −493 ppm, consistent with findings from previous studies on oxonium FESAN derivatives (Figure S10).^67^ In contrast, **BDP-COS** displays a typical pattern of 1:1:1:1:2:3:3:2:2:1:1 within the range of 24.8 to −28.9 ppm (Figure S14),^66^ while **BDP-B**_**12**_ exhibits four resonances ranging from 5.96 to −22.07 ppm (Figure S18).^65^ As expected, all compounds present a triplet between −0.65 and −0.46 ppm, corresponding to the boron atom of the BODIPY core.

### 2.2 Photophysical Properties

All compounds demonstrated high solubility in common organic solvents (acetone, THF, CH_3_CN, DMSO). Their photophysical properties were analyzed via UV–vis absorption and fluorescence emission spectroscopy in acetonitrile (ACN) at room temperature. UV–vis absorption spectra exhibited similar profiles across compounds, showing a main absorbance band in the visible region corresponding to the lowest-energy π–π* electronic transition of their BODIPY core (Figure 2, Table 1). However, iodination of the BODIPY core and subsequent alkylation of the *meso*-pyridyl group to introduce the boron clusters had some impact on UV–vis absorption (Table 1). As previously reported,^19^ a 35 nm bathochromic shift and a ca. 10% decrement in absorptivity coefficient at the absorbance maximum was observed for **BDP-I**_**2**_ (λ_abs,max_ = 535 nm) relative to noniodinated **BDP** (λ_abs,max_ = 500 nm). In addition, these two effects were further accentuated for **BDP-FES**, **BDP-COS** and **BDP-B**_**12**_ bearing a 4-pyridinium substituent at the *meso* position of the BODIPY chromophore, whose absorption maximum red-shifted up to λ_abs,max_ = 545 nm, as previously reported for similar compounds.^68^

**Figure 2:**
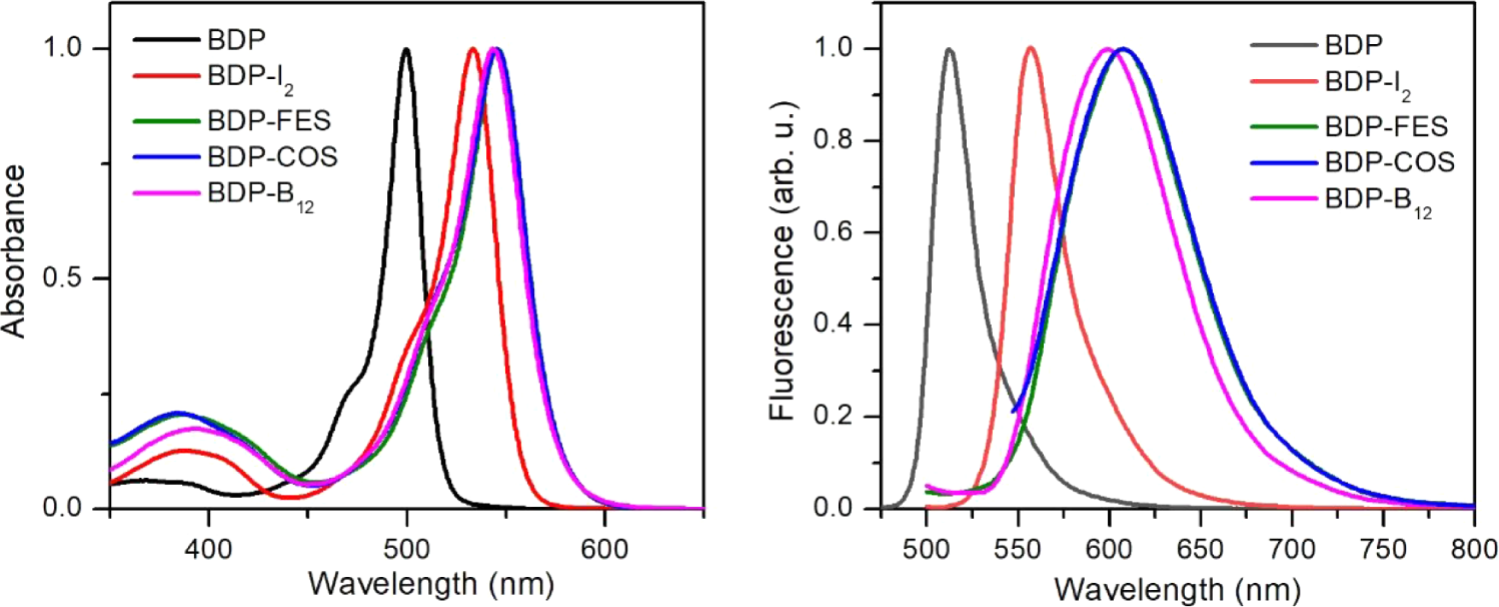
Absorption (left) and emission (right) spectra of all BODIPY derivatives prepared in ACN (*c* = 2.7 × 10^–6^ M), which have been normalized to unity at their maxima. The emission spectra were recorded at λ_exc_ = 490 nm.

**Table 1:** Photophysical Properties of Conjugates

Compound	*λ*_abs,max_ (nm)	ε_abs,max_ (M^–1^ cm^–1^)	*λ*_f,max_ (nm)	Φ_f_ ^a^	*k*_obs_/*k*_RB_ ^b^
**BDP**	500	85700	513	0.79	0.01
**BDP-I**_**2**_	535	78047	555	0.09	1.7
**BDP-FES**	545	51586	610	0.001	0.02
**BDP-COS**	545	43787	611	0.04	0.08
**BDP-B**_**12**_	545	49065	600	0.04	0.08

a Determined in acetonitrile solution at λ_exc_ = 490 nm, using rhodamine 6G as reference (Φ_f_ = 0.95 in EtOH).

b Apparent rate constant of photodegradation of a singlet oxygen chemical probe (DMA) in acetonitrile relative to Rose Bengal.

Much larger differences were encountered between these compounds when analyzing their emission properties (Figure 2, Table 1). Although all of them exhibited emission spectra that are consistent with BODIPY fluorescence, their emission maxima also bathochromically shifted from **BDP** (λ_f,max_ = 513 nm) to **BDP-I**_**2**_ (λ_f,max_ = 555 nm) and, especially, **BDP-FES**, **BDP-COS** and **BDP-B**_**12**_ (λ_f,max_ = 600–611 nm). As a result, very large Stokes shifts were measured for PSs bearing the boron clusters (45–66 nm), which could be attributed to the effect of the cationic *meso*-4-pyridinium attached to their BODIPY core. Despite this, the most striking difference registered between the fluorescent behavior of **BDP** and that of the rest of the compounds was related to their emission quantum yields (Φ_f_). Whereas **BDP** showed the high fluorescence efficiency expected for BODIPY emitters (Φ_f_ = 0.79), their iodinated derivatives presented markedly reduced Φ_f_ values (Φ_f_ = 0.001–0.09). For **BDP-I**_**2**_, **BDP-COS**, and **BDP-B**_**12**_ exhibiting similar fluorescence efficiencies (Φ_f_ = 0.04–0.09), this result can be mainly attributed to the heavy atom effect arising from iodination, as previously described for related iodinated BODIPY derivatives.^69,70^ Introduction of iodine atoms to the chromophore strengthens spin–orbit coupling and reduces the singlet–triplet energy gap (Δ*E*_ST_ ≈ 0.1 eV), thereby favoring nonradiative intersystem crossing (ISC) to the excited triplet states over radiative fluorescence from S_1_.^71–73^ In case of **BDP-FES** showing more profound suppression of its fluorescence emission, another factor should account for its very low Φ_f_ value (Φ_f_ = 0.001). By means of cyclic voltammetry measurements, we determined that the FESAN unit of this compound could be much easier reduced and oxidized than its BODIPY core, implying that the HOMO and LUMO orbitals of **BDP-FES** should lie on the boron cluster instead of on the chromophore (Figure S19). As a result, photoinduced electron transfer (PET) between its electronically excited BODIPY emitter and the FESAN unit should be highly favored for **BDP-FES**, an additional nonradiative relaxation mechanism that should strongly quench fluorescence emission.

Next, we evaluated the singlet oxygen (^1^O_2_) generation capacity of the compounds prepared. This was achieved by monitoring the photooxidation kinetics of the ^1^O_2_ scavenger 9,10-dimethylanthracene (DMA),^74,75^ when mixed with each BODIPY derivative and exposed to visible light, which was selectively absorbed by the photosensitizer. By plotting changes in DMA concentration with irradiation time determined from UV–vis absorption data, we could assess the ^1^O_2_ production rate for each photosensitizer (Figure 3, Figures S20–S25). Experimental data could be well fitted with a simple monoexponential kinetics model to obtain the apparent rate constant of ^1^O_2_ generation (*k*_obs_) for all compounds tested, which was then normalized using Rose Bengal (RB) as a standard (*k*_obs_/*k*_RB_, Table 1), which is regarded as one of the most effective producers of ^1^O_2_ upon irradiation.^76^

**Figure 3:**
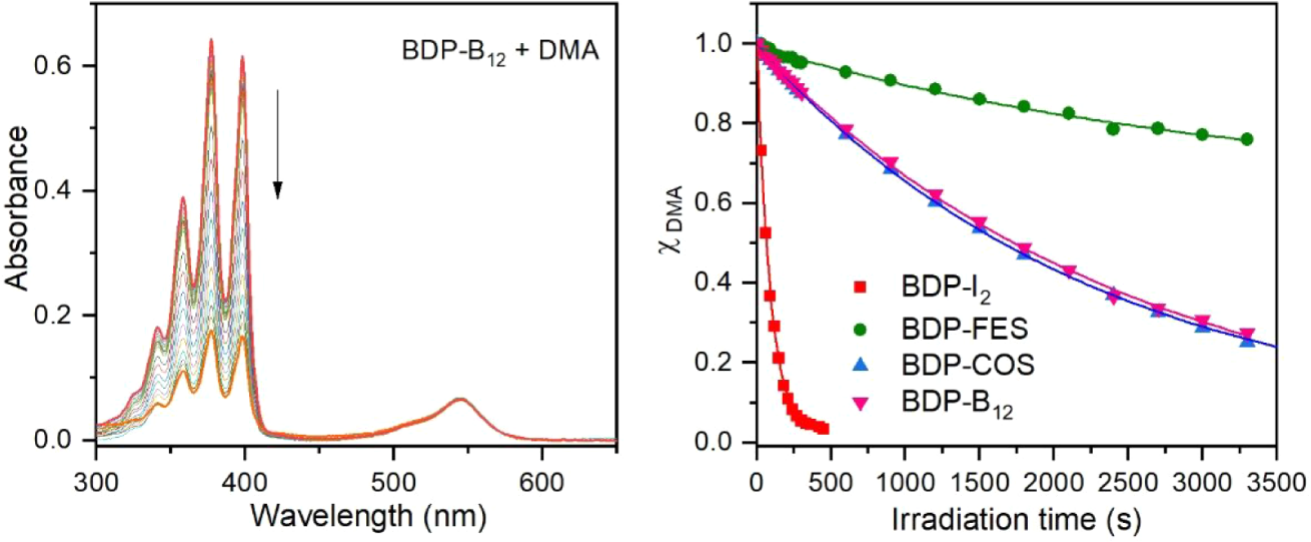
(left) Variation of the absorption spectra of a mixture of DMA (*c* = 6.0 × 10^–5^ M) and **BDP-B**_**12**_ (*c* = 2.7 × 10^–6^ M) in acetonitrile upon irradiation with a white light LED through a 435LP filter (power = 15 mW cm^–2^, λ_exc_ > 435 nm, irradiation from 0 to 3300 s). (right) DMA photoxidation kinetics measured for mixtures of DMA (*c* = 6.0 × 10^–5^ M) and all the iodinated BODIPY derivatives synthesized (*c* = 2.7 × 10^–6^ M) in acetonitrile. Symbols represent the experimental variation of the molar fraction of DMA (χ_DMA_) with the irradiation time for each mixture, while solid lines are fits to a monoexponential decay model.

As anticipated by its high fluorescence quantum yield and low ISC efficacy, BDP demonstrated low efficiency in producing ^1^O_2_ (*k*_obs_/*k*_RB_ = 0.01). In contrast, **BDP-I**_**2**_ exhibited high ^1^O_2_ generation rate, which was found to be even 1.7-fold larger than for RB at our experimental conditions. This result highlights the vital role of the iodine atoms introduced at the 2,6-position of the BODIPY core, which promote effective population of the triplet state through ISC and, as a consequence, ^1^O_2_ production by energy transfer to ground-state molecular oxygen ^3^O_2_). In spite of this, lower ^1^O_2_ generation efficiencies were determined for the photosensitizers where the diiodinated BODIPY chromophore was attached to boron clusters. For **BDP-FES**, this behavior can be attributed to preferent relaxation of its electronically excited singlet state via PET, which should not only quench fluorescence emission but also disfavor triplet state generation by ISC, thereby leading to low ^1^O_2_ generation efficiency (*k*_obs_/*k*_RB_ = 0.02). In case of **BDP-COS** and **BDP-B**_**12**_, the decrement in ^1^O_2_ production rate relative to **BDP-I**_**2**_ must be associated to their cationic *meso*-4-pyridinium substituent, as already been reported for other iodinated BODIPY dyes.^68^

### 2.3 Biological Activity

Due to their ability to generate singlet oxygen, **BDP-B**_**12**_ and **BDP-COS** were selected to evaluate their antimicrobial activity against various pathogens. Additionally, the parent compound **BDP-I**_**2**_ was also included in the evaluation.

#### 2.3.1 Evaluation of Antimicrobial Properties

To assess the antimicrobial activity of the selected photosensitizers against planktonic bacteria, we determined the minimum inhibitory concentration (MIC) for both Gram-positive species—*Staphylococcus aureus*, *Enterococcus faecium*, and *Enterococcus raffinosus*—and Gram-negative species—*Escherichia coli*, *Pseudomonas aeruginosa*, and *Enterobacter aerogenes*. None of them presented antibacterial or bactericidal activity in dark conditions (Tables 2 and 3).

**Table 2:** Minimal Inhibitory Concentrations (MICs) of PSs (in μM) against *E. coli*, *P. aeruginosa*, *E. aerogenes*, *S. aureus*, *E. faecium* and *E. raffinosus* ^a^

	BDP-COS		BDP-B_12_		BDP-I_2_	
	Dark	Light	Dark	Light	Dark	Light
*E. coli* ATCC 9637	>80	>80	>80	>80	>80	>80
*P. aeruginosa* PAO1	>80	>80	>80	>80	>80	>80
*E. aerogenes* CECT 684	>80	>80	>80	>80	>80	>80
*S. aureus* Newman	>80	>80	>80	**1.25**	>80	**0.15–<0.07**
*E. faecium* Efm5	>80	**2.5–5.0**	>80	**0.62**	>80	**<0.07**
*E. raffinosus* SWI	>80	**1.2–2.5**	>80	**0.31–0.62**	>80	**<0.07**

a The light dose was 28.2 J/cm^2^ for **BDP-B_12_** and **BDP-I_2_**, 84.6 J/cm^2^ for **BDP-COS**. Results are duplicates of at least three independent experiments.

**Table 3:** Bactericidal Activity Induced by Photodynamic Treatment (CFUs/mL) in *E. coli*, *P. aeruginosa*, *E. aerogenes*, *S. aureus*, *E. faecium* and *E. raffinosus*.^a^ ^b^

	BDP-COS		BDP-B_12_		BDP-I_2_	
	Dark	Light	Dark	Light	Dark	Light
*E. coli* ATCC 9637	UNC	UNC	UNC	UNC	UNC	UNC
*P. aeruginosa* PAO1	UNC	UNC	UNC	UNC	UNC	UNC
*E. aerogenes* CECT 684	UNC	UNC	UNC	UNC	UNC	UNC
*S. aureus* Newman	UNC	**0**	UNC	**0**	UNC	**0**
*E. faecium* Efm5	UNC	**0–10** ^**3**^	UNC	**0–10** ^**4**^	UNC	**0**
*E. raffinosus* SWI	UNC	**0–10** ^**3**^	UNC	**0**	UNC	**0**

a Colony-forming units/mL (CFUs/mL). UNC: uncountable colony-forming units.

b The light dose was 24.5 J/cm^2^ for **BDP-B**_**12**_ and **BDP-I**_**2**_, and 84.6 J/cm^2^ for **BDP-COS**. Results are based on triplicate measurements from a minimum of three independent experiments.

PSs exhibited a remarkable inhibitory activity in Gram-positive bacteria after exposure to green LED light (λ_max_ = 530 nm). **BDP-COS** had activity in *E. faecium* (MIC 2.5–5.0 μM) and *E. raffinosus* (MIC 1.2–2.5 μM), but not in *S. aureus* (Table 2). Remarkably, **BDP-B**_**12**_ was active against *S. aureus* (MIC 1.2 μM), as well as against *E. faecium* (MIC 0.62 μM) and *E. raffinosus* (MIC 0.31–0.62 μM) (Table 2). Parent **BDP-I**_**2**_ was also tested and showed activity against *S. aureus* (MIC 0.15–<0.07 μM) along with *E. faecium* and *E. raffinosus* (MIC < 0.07 μM) (Table 2).

The bactericidal effect was further evaluated by quantifying cell viability, observing a potent bactericidal response in Gram-positive bacteria. Although **BDP-COS** did not show bactericidal properties against *S. aureus* in MIC tests under illumination (Table 2), it instead led to no viable cells being recovered after light treatment in photoinactivation assays at 40 μM (Table 3). More probably, this discrepancy arises from the different analysis procedures used in the two methodologies applied. Thus, while immediate quantification of viable bacteria postirradiation was conducted in photoinactivation experiments, MIC assays involved a 24 h incubation period after light treatment, which could allow the growth of the few surviving cells. In addition, photoinactivation tests also revealed the bactericidal effect of **BDP-COS** against *E. faecium* and *E. raffinosus* (Table 3). With **BDP-B**_**12**_ no viable cells were detected in *S. aureus* and *E. raffinosus* and a reduction of 4 orders of magnitude or complete cell killing was observed in *E. faecium* (Table 3). Finally, no viable cells were recovered in the Gram-positive bacteria tested when parent **BDP-I**_**2**_ was evaluated (Table 3).

In general, between the two **BDP-I**_**2**_-derived compounds, **BDP-B**_**12**_ showed the best results against Gram-positive bacteria following light exposure. In *S. aureus*, dose–response analysis further supported the stronger bactericidal effect of **BDP-B**_**12**_ over **BDP-COS**, as it fully suppressed bacterial viability at the lowest tested concentration of 5 μM (Figure 4B), while **BDP-COS** exhibited a dose-dependent reduction with effects observed from 10 μM (Figure 4A). In addition, it is important to note that although **BDP-B**_**12**_ exhibited much lower singlet oxygen generation than **BDP-I**_**2**_, the former showed comparable bactericidal activity in *S. aureus* (Figure 4C).

**Figure 4:**
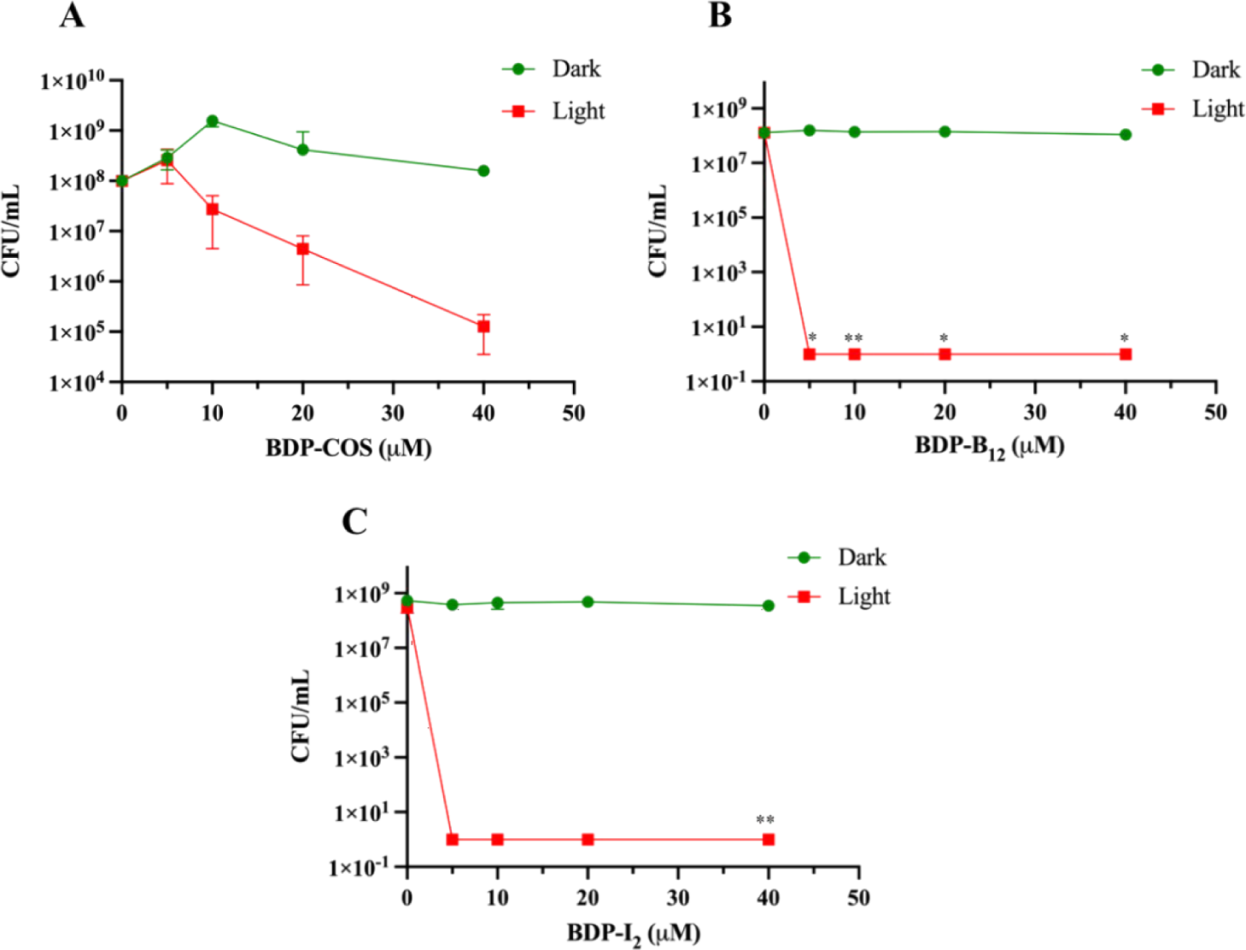
Dose–response curve of *S. aureus*, comparing the effect of light and darkness on the activity of the photosensitizers (A) **BDP-COS**, (B) **BDP-B**_**12**_ and (C) **BDP-I**_**2**_. The light dose was 28.2 J/cm^2^ for **BDP-B**_**12**_ and for **BDP-I**_**2**_, and 84.6 J/cm^2^ for **BDP-COS**. Data represents the mean of three replicates. The detection limit was 1 CFU/mL. Statistical significance: *p < 0.01 ** *p* < 0.001.

The results obtained with the parent compound **BDP-I**_**2**_ showed antibacterial and bactericidal activities against the Gram-positive strains comparable with **BDP-B**_**12**_ (Tables 2 and 3); however, **BDP-I**_**2**_ exhibited much lower solubility in aqueous solutions. This limitation underscores the importance of developing the functionalized derivatives, which would not only improve water solubility but also hold promise for enhanced performance and broader applicability in biological settings. It is also important to note that, although **BDP-B**_**12**_ exhibited much lower singlet oxygen generation than **BDP-I**_**2**_, the former showed comparable antibacterial efficacy, which could suggest a greater interaction with the bacterial membrane. On the other hand, none of PSs had any effect on Gram-negative bacteria after light exposure. The lack of activity in Gram-negative bacteria could be related to the nature of their cell wall, in terms of surface charge, hydrophobicity and structure.^77^ The outer membrane acts as a selective permeability barrier that may interfere with the uptake of PSs, compromising their ability to reach intracellular targets.,^78,79^ Similar results have been observed with some **BODIPY**-derivate PSs showing no activity against *Klebsiella pneumoniae* ^80^ or *P. aeruginosa*.^81^

## 3 Conclusions

Three novel iodinated anionic boron cluster-BODIPY conjugates, **BDP-FES** (BODIPY-ferrabisdicarbollide), **BDP-COS** (BODIPY-cobaltabisdicarbollide), and **BDP-B**_**12**_ (BODIPY-*closo*-dodecaborate), have been designed to enhance antimicrobial photodynamic therapy (aPDT) against drug-resistant pathogens. Functionalization of the iodinated BODIPY (**BDP-I**_**2**_) with the different ionic boron clusters induced a red-shift both in the absorption and emission maxima, as well as an important suppression of the fluorescence quantum yields. This result was attributed to the heavy-atom promoted generation of excited triplet states, as well as photoinduced electron transfer mainly in **BDP-FES**. Crucially, these molecular changes enabled selective fine-tuning of singlet oxygen generation and enhanced water solubility, key parameters for optimizing photosensitizer performance. Remarkably, both conjugates **BDP-B**_**12**_ and **BDP-COS** exhibited potent light-dependent bactericidal activity against Gram-positive pathogens (*S. aureus*, *E. faecium*, *E. raffinosus*) but no intrinsic toxicity in darkness. Among the conjugates, **BDP-B**_**12**_ emerged as the most promising photosensitizer, achieving full eradication of *S. aureus* at just 5 μM under green light exposure. Despite generating lower singlet oxygen levels than its iodinated precursor **BDP-I**_**2**_, **BDP-B**_**12**_ demonstrated comparable or superior bactericidal efficacy, suggesting that effective antimicrobial action can be achieved not only by maximizing reactive oxygen species production, but also by optimizing molecular structure. Both **BDP-B**_**12**_ and **BDP-COS** showed no intrinsic toxicity in darkness, confirming their selective activation and safety profile. No activity was observed against Gram-negative bacteria, attributed to cell-wall barriers. Notably, the conjugates offered significantly better water solubility than the parent compound (**BDP-I**_**2**_), addressing a critical limitation for feasible application. Our findings validate anionic boron clusters as versatile and effective structural motifs to modulate the photophysical and biological properties of BODIPY-based photosensitizers, improving water solubility and pathogen selectivity, key factors for practical application in antimicrobial surface disinfection and infection control. This work serves as the first framework for deploying boron-cluster–BODIPY hybrids in aPDT, bridging inorganic synthesis and applied PDT with translational biomedical relevance. Future studies will be directed toward optimizing biofilm penetration, expanding activity toward Gram-negative pathogens, and evaluating *in vivo* efficacy and safety to advance these conjugates toward real-world applications.

## 4 Experimental Section

### 4.1 Reagents and Materials

All reactions were performed under atmosphere of dinitrogen employing standard Schlenk techniques. All reagents were purchased and used without further purification. Acetonitrile was dried for 2 days over molecular sieves (3Å; Merck; activated for 16h at 300 °C under vacuum). After drying, acetonitrile was degassed using the well stablished freeze–pump–thaw protocol. Reactions were monitored by TLC on precoated silica gel plates (ALUGRAM SILG/UV254) and revealed by exposure to a UV254 lamp. Flash column chromatography was performed using silica gel (230–400 mesh). Commercial grade tetrahydrofuran, methanol, ethanol, hexane, dichloromethane and acetone were used without further purification. Compounds *closo-*dodecaborate-dioxane,^65^ COSAN-dioxane,^66^ and FESAN-dioxane^67^ were synthesized according to the literature (Supporting Information).

### 4.2 Instrumentation

Infrared spectra were recorded on a JASCO FT-IR spectrophotometer, and wavenumber is reported in cm^–1^. Elemental analyses were obtained using a Thermo (Carlo Erba) Flash 2000 Elemental Analyzer, configured for wt % CHN. The ^1^H NMR (300.13 MHz), ^11^B {^1^H} (96.29 MHz), ^13^C {^1^H} NMR (75.47 MHz) and ^19^F NMR (282.4 MHz) spectra were recorded on a Bruker DPX300 (300 MHz) spectrometer in CDCl_3_ and acetone-d_6_ at room temperature. Chemical shifts are reported in ppm using the signal of the residual nondeuterated solvent molecules as reference for ^1^H and ^13^C NMR. For ^11^B­{^1^H} NMR spectra were referenced to external BF_3_.OEt_2_. All coupling constants are reported in hertz. UV–vis absorption spectra were recorded on a JASCO V-770 UV–vis spectrophotometer or on a Agilent HP 8453 spectrophotometer using 1 cm cuvettes, and the emission spectra were measured on an FS5 spectrofluorometer from Edinburgh Instruments. Samples were prepared in spectroscopic grade solvents and adjusted to a response within the linear range. No fluorescent contaminants were detected on excitation in the wavelength region of experimental interest. The fluorescence quantum yields of all compounds were calculated by comparison using Rhodamine 6G in EtOH (Φ_f_ = 0.95) as a standard, and corrected for the refractive index of the solvent. Samples were prepared in such a way as to obtain an absorbance around 0.1 at the excitation wavelength. 9,10-dimethylanthracene (DMA) was used as a chemical trap to investigate the singlet oxygen photosensitizing properties of all PSs. With this aim, mixtures of DMA and conjugates were prepared in ACN (*c*_DMA_ = 6.0 × 10^–5^ M, *c*_PS_ = 2.7 × 10^–6^ M) and the variation of their absorption spectra upon visible light irradiation was measured in time. In these experiments, selective irradiation of the complexes was accomplished by using a 435LP long-pass filter to spectrally remove the violet component of a white light LED source (power = 15 mW cm^–2^, as measured with a calibrated power meter (Gentec TPM-300) placed at the same position as the sample and after the long-pass filter). Control experiments were also conducted to investigate the photostability of DMA under equivalent illumination conditions as well as the stability of the mixtures of DMA and conjugates in the dark. We conducted irradiation experiments on bacterial cultures using a Cameo STUDIO PAR 6 G2 LED spotlight as the source of irradiation, specifically utilizing only its green emission channel. To determine the light dose (J/cm^2^) the fluence rate (irradiance) of the green LED source was measured using a calibrated optical power meter (LASERPOINT PC-PLUG A-2-D12-HPB-U, sensitivity 286.57 mV–7 W). The light dose was then calculated according to the following formula: Dose (J/cm^2^) = Fluence rate (mW/cm^2^) × Time (s) × 10^–3^. Measurements were performed by positioning the LED source above the bacteria-containing plate at a distance to ensure a homogeneous irradiance.

### 4.3 Synthesis and Characterization of Compounds

#### 4.3.1 Synthesis of **BDP**

To synthesize **BDP**, 1 g (9.34 mmol) of pyridine-4-carbaldehyde was added to a 250 mL round-bottom flask and dissolved in 40 mL of anhydrous THF. Under N_2_ atmosphere, 2 mL (19.61 mmol) of 2,4-dimethylpyrrole and 286 μL (3.73 mmol) of trifluoroacetic acid were added. The reaction mixture was left stirring for 10 h at room temperature. Afterward, the inert atmosphere was removed, and 2.12 g (9.34 mmol) of 1,3-dichloro-5,6-dicyano-1,4-benzoquinone (DDQ) was added. This oxidation was conducted for 12 h. Then, the round-bottom flask was placed in an ice bath, and 19.5 mL (140.04 mmol) of triethylamine (TEA) was added. After 10 min, 17.3 mL (140.04 mmol) of boron trifluoride diethyl etherate (BF_3_·OEt_2_) was added dropwise into the reaction flask. The reaction was left to react for 3 h at room temperature. Then, the THF was removed under reduced pressure until a brown viscous mixture was obtained, and then it was redissolved in 75 mL of dichloromethane (DCM). The solution was divided into three fractions of 25 mL; each was washed with 25 mL(×3) of distilled water. Once all the organic phases were dried with MgSO_4_ and recollected, the volatiles were evaporated under reduced pressure, and the crude product was supported on silica gel and purified by column chromatography using silica gel as a stationary phase with hexane:ethyl acetate (7:3) as eluent to give **BDP** as an orange solid (700 mg, 23% yield for three steps). UV–vis (ACN): λ_max_ = 500 nm. ^1^H NMR (300 MHz, CO­(CD_3_)_2_, δ, ppm): 8.86 (dd, *J*_1_ = 4.4 Hz, *J*_2_ = 1.5 Hz, 2H, pyridyl), 7.54 (dd, *J*_1_ = 4.4 Hz, *J*_2_ = 1.5 Hz, 2H, pyridyl), 6.20 (s, 2H, pyrrolyl), 2.56 (s, 6H, methyl), 1.48 (s, 6H, methyl). ^13^C NMR (75 MHz, CO­(CD_3_)_2_, δ, ppm): 156.0 (pyridyl), 150.7 (pyridyl), 142.9 (pyrrolyl), 142.8 (pyrrolyl), 138.7 (pyrrolyl), 130.3 (meso), 123.3 (pyridyl), 121.6­(pyrrolyl), 13.8 (methyl), 13.7 (methyl). ^19^F (282.4 MHz, CO­(CD_3_)_2_, δ, ppm): −145.7 (q, *J*_B–F_ = 32.2 Hz). ^11^B (96.3 MHz, CO­(CD_3_)_2_, δ, ppm): 0.65 (t, *J*_B–F_ = 32.2 Hz).

#### 4.3.2 Synthesis of **BDP-I**_**2**_

To synthesize **BDP-I**_**2**_, 100 mg (0.308 mmol) of **BDP,** 468.3 mg (1.85 mmol) of iodine (I_2_), and 324.6 mg (1.85 mmol) of iodic acid (HIO_3_) were combined in a round-bottom flask and dissolved in 35 mL of absolute ethanol. The reaction mixture was stirred at room temperature for 18 h. After this period, the ethanol was evaporated under reduced pressure. The resulting crude reaction mixture was then dissolved in 30 mL of dichloromethane and washed twice with 30 mL of saturated aqueous sodium thiosulfate (Na_2_S_2_O_3_) solution. The organic phase was dried over magnesium sulfate (MgSO_4_), and the solvent was again evaporated under reduced pressure. Finally, the crude product was adsorbed onto silica gel and purified by column chromatography using silica gel as the stationary phase with a hexane: ethyl acetate: triethylamine (8:1.95:0.05) eluent system. **BDP-I**_**2**_ was obtained as a red solid (89 mg, 50% yield). UV–vis (ACN): λ_max_ = 535 nm. ^1^H NMR (300 MHz, CDCl_3_, δ, ppm): 8.83 (d, *J* = 4.5 Hz, 2H, pyridyl), 7.30 (d, *J* = 4.5 Hz, 2H, pyridyl), 2.67 (s, 6H, methyl), 1.44 (s, 6H, methyl). ^13^C NMR (75 MHz, CDCl_3_, δ, ppm): 157.8 (pyridyl), 150.9 (pyridyl). 144.8 (pyrrolyl), 143.3 (pyrrolyl), 137.1 (pyrrolyl), 130.2 (meso), 123.0 (pyridyl), 86.4 (iodinated), 17.2 (methyl), 16.1 (methyl). ^19^F (282.4 MHz, CO­(CD_3_)_2_, δ, ppm): −145.7 (q, *J*_B–F_ = 32.2 Hz). ^11^B (96.3 MHz, CO­(CD_3_)_2_, δ, ppm): 0.65 (t, *J*_B–F_ = 32.2 Hz).

#### 4.3.3 Synthesis of **BDP-FES**

To synthesize **BDP-FES**, a 10 mL two-necked dry round-bottom flask equipped with a condenser and a magnetic stir bar was prepared. Inside the flask, 25 mg (0.043 mmol) of **BDP-I**_**2**_ and 21 mg (0.052 mmol) of FESAN–dioxane were placed and dried under vacuum at room temperature for 10 min using the Schlenk line. Under a nitrogen atmosphere, 5 mL of anhydrous acetonitrile was added to the flask. The mixture was stirred and heated under reflux for 24 h. After the reaction, the mixture was cooled to room temperature and adsorbed onto silica gel. The solvent was evaporated under reduced pressure, and the crude product was purified by column chromatography. Silica gel was used as the stationary phase, and the eluent system consisted of dichloromethane and methanol in a ratio of 98:2 to give **BDP-FES** as a violet solid (25 mg, 59% yield). UV–vis (ACN): λ_max_ = 545 nm. Elemental analysis calcd. (%) for C_26_H_41_B_19_FeF_2_I_2_N_3_O_2_·0.7C_6_H_14_: C 34.84, H 4.92, N 4.04; found: C 35.13, H 5.13, N 3.96. IR (ATR, cm^–1^): 3057 (w), 21916 (w), 2856 (w), 2537 (s), 1637 (s), 1529 (s), 1452 (m), 1342 (m), 1174 (s), 994 (m). ^1^H NMR (300 MHz, CO­(CD_3_)_2_, δ, ppm): 73.37 (br, cluster), 58.53 (br, cluster), 43.75 (br, cluster), 40.19 (br, cluster), 27.09 (br, cluster), 5.28 (s, 2H, cluster), 3.64 (s, 4H, cluster), 3.06 (s, 2H, cluster), 2.30 (s, 6H, methyl), 1.46 (s, 4H, ethylene glycol chain), −0.43 (s, 2H, cluster), −1.23 (s, 6H, methyl), −4.64 (s, 2H, ethylene glycol chain), −9.73 (S, 2H, ethylene glycol chain), −11.71 (br, 2H, cluster). ^13^C NMR (75 MHz, CO­(CD_3_)_2_, δ, ppm): 157.7 (pyridyl), 149.0 (pyrrolyl), 143.2 (pyrrolyl), 141.9 (pyridyl), 132.1 (pyridyl), 127.9 (meso), 125.0 (pyridyl), 85.5 (iodinated), 67.2 5 (ethylene glycol chain), 60.5 (ethylene glycol chain), 56.2 (cluster), 25.3, 15.1 (methyl). ^19^F (282.4 MHz, CO­(CD_3_)_2_, δ, ppm): −145.7 (q, *J*_B–F_ = 31.2 Hz). ^11^B­{^1^H} (96.3 MHz, CO­(CD_3_)_2_, δ, ppm): 116.42 (s, 1B, B–H), 99.07 (s, 1B, B–H), 26.87 (s, 2B, B–H), 22.75 (s, 2B, B–H), −0.33 (t, *J*_B–F_ = 31.2, 1B, BF_2_), −1.07 (s, 3B, B–H), −5.23 (s, 3B, B–H), −34.34 (s, 1B, B–H), −37.42 (s, 1B, B–H), −376.54 (s, 1B, B–H), −401.32 (s, 1B, B–H), −441.00 (s, 1B, B–H), −492.54 (s, 1B, B–H).

#### 4.3.4 Synthesis of **BDP-COS**

This compound was obtained as described for **BDP-FES** using 25 mg (0.043 mmol) of **BDP-I**_**2**_, 21.2 mg (0.052 mmol) of COSAN-dioxane, and 5 mL of anhydrous acetonitrile. The crude product was purified using column chromatography with silica gel as the stationary phase and a dichloromethane: methanol (98:2) eluent system. **BDP-COS** was obtained as a violet solid (27 mg, 64% yield). UV–vis (ACN): λ_max_ = 545 nm. Elemental analysis calcd. (%) for C_26_H_41_B_19_CoF_2_I_2_N_3_O_2_·0.7C_6_H_14_: C 34.33, H 4.81, N 4.06; found: C 34.31, H 5.21, N 4.09. IR (ATR, cm^–1^): 3121 (w), 3056 (m), 2924 (m), 2854 (w), 2536 (s), 2359 (m), 1637 (m), 1530 (s), 1455 (m), 1308 (m), 1172 (s), 994 (m). ^1^H NMR (300 MHz, CO­(CD_3_)_2_, δ, ppm): 9.63 (d, *J* = 6.8 Hz, 2H, pyridyl), 8.64 (d, *J* = 6.8 Hz, 2H, pyridyl), 5.20 (t, *J* = 4.7 Hz, 2H, ethylene glycol chain), 4.29 (t, *J* = 4.7 Hz, 2H, ethylene glycol chain), 4.11 (bs, 2H, cluster), 4.03 (bs, 2H, cluster), 3.75–3.67 (m, 4H, ethylene glycol chain), 2.68 (s, 6H, methyl), 1.61 (s, 6H, methyl). ^13^C NMR (75 MHz, CO­(CD_3)2_, δ, ppm): 158.5 (pyridyl), 152.2 (pyrrolyl), 147.3 (pyridyl),144.9 (pyrrolyl), 134.2 (pyrrolyl), 129.5 (meso), 128.5 (pyridyl), 86.5 (iodinated), 72,6, 69.2, 69.2, 62.3 (ethylene glycol chain), 52.0 (cluster), 46. Five (cluster), 17.4 (methyl), 15.5 (methyl). ^19^F (282.4 MHz, CO­(CD_3_)_2_, δ, ppm): −145.7 (q, *J*_B–F_ = 31.4 Hz). ^11^B­{^1^H} (96.3 MHz, CO­(CD_3_)_2_, δ, ppm): 24.77 (s, 1B, B–O), 6.60 (s, 2B, B–H), 0.46 (t, *J*_B–F_ = 31.4, 2B, BF_2_, B–H), −2.71 (s, 1B, B–H), −4.82 (s, 1B, B–H), −6.83 (s, 5B, B–H), −8.82 (s, 1B, B–H), −17.29 (s, 2B, B–H), −19.97 (s, 2B, B–H), −22.37 (s, 1B, B–H), −28.87 (s, 1B, B–H).

#### 4.3.5 Synthesis of **BDP-B**_**12**_

This compound was obtained as described for **BDP-FES** using 25 mg (0.043 mmol) of **BDP-I**_**2**_, 98.0 mg (0.208 mmol) of *closo*-dodecaborate-dioxane, and 6 mL of anhydrous acetonitrile. The crude product was purified using column chromatography with silica gel as the stationary phase and a dichloromethane: methanol (92:8) eluent system. **BDP-B**_**12**_ was obtained as a violet solid (7 mg, 15% yield). UV–vis (ACN): λ_max_ = 545 nm. Elemental analysis calcd. (%) for C_38_H_71_B_13_F_2_I_2_N_4_O_2_·2.1C_7_H_16_: C 50.29, H 8.38, N 4.45; found: C 50.56, H 8.00, N 4.27. IR (ATR, cm^–1^): 3172 (w), 3056 (w), 2960 (m), 2928 (m), 2871 (m), 2520 (s), 2362 (s), 1637 (w), 1521 (m), 1458 (m), 1342 (m), 1177 (s), 993 (m). ^1^H NMR (300 MHz, CO­(CD_3_)_2_, δ, ppm): 9.96 (d, *J* = 6.8 Hz, 2H, pyridyl), 8.40 (d, *J* = 6.8 Hz, 2H, pyridyl), 5.22 (t, *J* = 4.1 Hz, 2H, ethylene glycol chain), 4.12 (t, *J* = 4.1 Hz, 2H, ethylene glycol chain), 3.76–3.72 (m, 2H, ethylene glycol chain), 3.69–3.64 (m, 2H, ethylene glycol chain), 3.49–3.41 (m, 8H, ^+^NBu_4_), 2.63 (s, 6H, methyl), 1.89–1.78 (m, 8H, ^+^NBu_4_), 1.58 (s, 6H, methyl), 1.51–1.38 (m, 8H, ^+^NBu_4_), 0.99 (t, *J* = 7.3 Hz, 12H, ^+^NBu_4_). ^13^C NMR (75 MHz, CO­(CD_3)2_, δ, ppm): 158.2 (pyridyl), 150.9 (pyrrolyl), 148.8 (pyridyl), 145.2 (pyrrolyl), 135.0 (pyrrolyl), 129.5 (pyrrolyl), 127.8 (pyridyl), 86.5 (iodinated), 74.1, 69.4, 67.4, 61.6 (ethylene glycol chain), 58.8, 23.6, 19.5 (^+^NBu_4_), 17.6, 15.6 (methyl-Bodipy), 13 (^+^NBu_4_). ^19^F (282.4 MHz, CO­(CD_3_)_2_, δ, ppm): −145.7 (q, *J*_B–F_ = 31.3 Hz). ^11^B­{^1^H} (96.3 MHz, CO­(CD_3_)_2_, δ, ppm): 5.96 (s, 1B, B–O), 0.47 (t, *J*_B–F_ = 31.3 Hz, 1B, BF_2_), −16.72 (s, 5B, B–H), −17.40 (s, 5B, B–H), −22.07 (s, 1B, B–H).

### 4.4 Biological Evaluation

#### 4.4.1 Preparation of Photosensitizers

Stocks solutions of the PSs were prepared at 10 mM in 100% DMSO. Intermediate solutions at 1 mM were prepared in distilled water, and working solutions were further diluted in culture medium.

#### 4.4.2 Microbial Strains and Growth Conditions

The strains used in this study were mostly retrieved from our own collection. *Staphylococcus aureus* Newman (ATCC 25904) and *Enterococcus raffinosus* SWI, an environmental isolate obtained from soil, were included. *Enterococcus faecium* Efm5, a clinical strain isolated from a patient with bacteremia at Parc Taulí University Hospital, was kindly provided by Dr. Óscar Quijada.^82^ These strains were selected as Gram-positive bacteria. *Escherichia coli* ATCC 9637, *Pseudomonas aeruginosa* PAO1 and *Enterobacter aerogenes* CECT 684 were selected as Gram-negative bacteria. For maintenance, *S. aureus* Newman, *E. coli* ATCC 9637, *P. aeruginosa* PAO1 and *E. aerogenes* CECT 684 were grown in Luria–Bertani (LB); *E. faecium* Efm5 and *E. raffinosus* SWI were grown in Brain Heart Infusion (BHI) agar. All cultures were incubated at 37 °C.

#### 4.4.3 Antimicrobial Susceptibility Testing

Minimum Inhibitory Concentration (MIC) was determined by microdilution method as previously described,^83^ and according to the Clinical and Laboratory Standards Institute European Committee on Antimicrobial Susceptibility Testing. Breakpoint tables for interpretation of MICs and zone diameters. Version 8.0 (2018). www.eucast.org/clinicalbreakpoints/. Briefly, overnight cultures were diluted in cation-adjusted Mueller–Hinton (CAMHB) broth to achieve a concentration of 1 × 10^6^ CFUs/mL (final concentration of 5 × 10^5^ CFUs/mL). 50 μL were then added to each well of a 96-well plate containing 50 μL of 2-fold serial dilutions of each compound in duplicate. PSs were added to obtain a maximum concentration of 80 μM from the working solution (1 mM) as was previously described.^68^ Sterility (no bacteria) and growth controls (no PSs, but the same amount of DMSO when the highest compound concentration was tested) were also included. Two plates were prepared: one for dark and the other one for green LED light exposition. A 12W “Cameo STUDIO Par 6 G2” LED light was used as a light source using a light dose of 28.2 J/cm^2^ for **BDP-I**_**2**_ and **BDP-B**_**12**_, and 84.6 J/cm^2^ for **BDP-COS**, with a maximum emission of 530 nm with an incubation time of 30 min in dark. After irradiation, plates were incubated at 37 °C for 24 h. Breakpoint values were determined by measuring the absorbance at 550 nm using a Multiskan FC plate reader (Thermo Fisher Scientific). MICs were interpreted as the PSs concentrations achieving ≥80% reduction in bacterial growth relative to the untreated controls. All the experiments were done in dark, avoiding artificial room light. Two technical replicates were made, and the experiment was performed at least three times.

#### 4.4.4 Photodynamic Inactivation and Dose–Response Assays

Photodynamic inactivation assays were performed as previously described,^68^ with some modifications. Overnight cultures of *S. aureus* Newman ATCC 25904, the clinical isolate of *E. faecium*, *E. raffinosus* SWI, *E. coli* ATCC 9637, *P. aeruginosa* PAO1 and *E. aerogenes* CECT 684, were diluted in CAMHB to obtain a final concentration of 1 × 10^8^ CFUs/mL. For the standard assay 50 μL of bacterial suspension were mixed with 50 μL of each PSs at final concentration of 40 μM in 96-well plates. To evaluate the effect of increasing concentration of PSs, dose–response assays were also conducted using *S. aureus* Newman. In this case, 50 μL of bacterial suspension were mixed with 50 μL of the PSs diluted in CAMHB to reach final concentrations of 40 μM, 20 μM, 10 μM, 5 μM and 0 μM. In all cases, two plates were prepared and incubated in dark at room temperature for 30 min after PS addition. One plate was then exposed to green LED light as previously described. The number of viable cells was determined by plating serial dilutions onto LB agar plates. Plates were incubated for 24 h at 37 °C. Three technical and biological replicates were made. The average of CFU/mL was evaluated. In the dose–response assay, the detection limit was 1 CFU/mL.

#### 4.4.5 Statistical Analyses

Statistical analyses were performed using GraphPad Prism v.9.0 software (San Diego, CA, USA). Two-way ANOVA test was used to evaluate the differences between groups.

## Supplementary Material



## Notes

Supplementary spectroscopic characterization: NMR spectra; additional electrochemical characterization: cyclic voltammograms and measurements of singlet oxygen production (PDF)

## References

[1] Laxminarayan R., Matsoso P., Pant S., Brower C., Røttingen J. A., Klugman K., Davies S. (2016). Access to Effective Antimicrobials:
A Worldwide Challenge. Lancet.

[2] GBD 2021 Antimicrobial Resistance Collaborators Global Burden of Bacterial Antimicrobial Resistance 1990 – 2021: A Systematic Analysis with Forecasts to 2050. Lancet. 2024,404, 1199-1226. 10.1016/S0140-6736(24)01867-1. 39299261 PMC11718157

[3] Kariuki S. (2024). Comment Global
Burden of Antimicrobial Resistance and Forecasts To. Lancet.

[4] Dube E. (2024). Antimicrobial
Photodynamic Therapy: Self-Disinfecting Surfaces for Controlling Microbial
Infections. Microorganisms.

[5] Mesquita M. Q., Dias C. J., Neves M. G. P. M. S., Almeida A., Faustino M. A. F. (2018). Revisiting
Current Photoactive Materials for Antimicrobial Photodynamic Therapy. Molecules.

[6] Li X., Bai H., Yang Y., Yoon J., Wang S., Zhang X. (2019). Supramolecular
Antibacterial Materials for Combatting Antibiotic Resistance. Adv. Mater..

[7] Cieplik F., Tabenski L., Buchalla W., Maisch T. (2014). Antimicrobial Photodynamic
Therapy for Inactivation of Biofilms Formed by Oral Key Pathogens. Front. Microbiol..

[8] Shi Q., Mou C., Xie Z., Zheng M. (2022). Photodiagnosis and Photodynamic Therapy
Exploring BODIPY Derivatives as Photosensitizers for Antibacterial
Photodynamic Therapy. Photodiagn. Photodyn.
Ther..

[9] Loudet A., Burgess K. (2007). BODIPY Dyes and Their Derivatives: Syntheses and Spectroscopic
Properties. Chem. Rev..

[10] Lu H., Shen Z. (2020). Editorial: BODIPYs
and Their Derivatives: The Past, Present and Future. Front. Chem..

[11] Ordóñez-Hernández J., Arcos-Ramos R., Alvarez-Venicio V., Basiuk V. A., González-Antonio O., Flores-Álamo M., García-Ortega H., Farfán N., Carreón-Castro M. D. P. (2021). Engineering Coumarin-BODIPY Thin-Films
and Molecular Crystals: Tailoring Supramolecular Self-Assembly for
Organic Electronic Applications. J. Mol. Struct..

[12] Li H., Wang J., Jiao L., Hao E. (2024). BODIPY-Based Photocages:
Rational Design and Their Biomedical Application. Chem. Commun..

[13] Das S., Dey S., Patra S., Bera A., Ghosh T., Prasad B., Sayala K. D., Maji K., Bedi A., Debnath S. (2023). BODIPY-Based
Molecules for Biomedical Applications. Biomolecules.

[14] Bumagina N. A., Antina E. V. (2024). Review of Advances
in Development of Fluorescent BODIPY
Probes (Chemosensors and Chemodosimeters) for Cation Recognition. Coord. Chem. Rev..

[15] Bellier Q., Pégaz S., Aronica C., Guennic B. L., Andraud C., Maury O. (2011). Near-Infrared Nitrofluorene Substitued Aza-Boron-Dipyrromethenes
Dyes. Org. Lett..

[16] Teresa V., Eleonora O., Fabrizio M., Enrico B. (2022). Searching for Antimicrobial
Photosensitizers among a Panel of BODIPYs. Photochem.
Photobiol. Sci..

[17] Wang D., Wang X., Zhou S., Gu P., Zhu X., Wang C., Zhang Q. (2023). Evolution of BODIPY as Triplet Photosensitizers
from Homogeneous to Heterogeneous: The Strategies of Functionalization
to Various Forms and Their Recent Applications. Coord. Chem. Rev..

[18] Hu W., Zhang R., Zhang X. F., Liu J., Luo L. (2022). Halogenated
BODIPY Photosensitizers: Photophysical Processes for Generation of
Excited Triplet State, Excited Singlet State and Singlet Oxygen. Spectrochim. Acta, Part A.

[19] Banfi S., Nasini G., Zaza S., Caruso E. (2013). Synthesis
and Photo-Physical
Properties of a Series of BODIPY Dyes. Tetrahedron.

[20] Caruso E., Gariboldi M., Sangion A., Gramatica P., Banfi S. S. (2017). Photodynamic Activity, and Quantitative Structure-Activity
Relationship Modelling of a Series of BODIPYs. J. Photochem. Photobiol., B.

[21] Grimes, R. N. Carboranes, 3r ed; Academic Press, 2016.

[22] Stockmann P., Gozzi M., Kuhnert R., Sárosi M. B., Hey-Hawkins E. (2019). New Keys for
Old Locks: Carborane-Containing Drugs
as Platforms for Mechanism-Based Therapies. Chem. Soc. Rev..

[23] Issa F., Kassiou M., Rendina L. M. (2011). Boron in
Drug Discovery: Carboranes
as Unique Pharmacophores in Biologically Active Compounds. Chem. Rev..

[24] Grams R. J., Santos W. L., Scorei I. R., Abad-García A., Rosenblum C. A., Bita A., Cerecetto H., Viñas C., Soriano-Ursúa M. A. (2024). The Rise of Boron-Containing
Compounds: Advancements in Synthesis, Medicinal Chemistry, and Emerging
Pharmacology. Chem. Rev..

[25] Fink K., Uchman M. (2021). Boron Cluster Compounds
as New Chemical Leads for Antimicrobial
Therapy. Coord. Chem. Rev..

[26] Gutiérrez-Gálvez L., García-Mendiola T., Lorenzo E., Nuez-Martinez M., Ocal C., Yan S., Teixidor F., Pinheiro T., Marques F., Viñas C. (2024). Compelling
DNA Intercalation through
‘Anion-Anion’ Anti-Coulombic Interactions: Boron Cluster
Self-Vehicles as Promising Anticancer Agents. J. Mater. Chem. B.

[27] Núñez R., Romero I., Teixidor F., Viñas C. (2016). Icosahedral
Boron Clusters: A Perfect Tool for the Enhancement of Polymer Features. Chem. Soc. Rev..

[28] Núñez R., Tarrés M., Ferrer-Ugalde A., De Biani F. F., Teixidor F. (2016). Electrochemistry
and Photoluminescence of Icosahedral Carboranes, Boranes, Metallacarboranes,
and Their Derivatives. Chem. Rev..

[29] Romero I., Martinez-Medina M., Camprubí-Font C., Bennour I., Moreno D., Martínez-Martínez L., Teixidor F., Fox M. A., Viñas C. (2020). Metallacarborane
Assemblies as Effective Antimicrobial Agents, Including a Highly Potent
Anti-MRSA Agent. Organometallics.

[30] Bennour I., Ramos M. N., Nuez-Martinez M., Xavier J. A. M., Buades A. B., Sillanpaa R., Teixidor F., Choquesillo-Lazarte D., Romero I., Martinez-Medina M., Viñas C. (2022). Water Soluble
Organometallic Small Molecules as Promising Antibacterial Agents:
Synthesis, Physical-Chemical Properties and Biological Evaluation
to Tackle Bacterial Infections. Dalton Trans..

[31] Kubiński K., Masłyk M., Janeczko M., Goldeman W., Nasulewicz-Goldeman A., Psurski M., Martyna A., Boguszewska-Czubara A., Cebula J., Goszczyński T.
M. (2022). Metallacarborane Derivatives
as Innovative Anti- Candida Albicans Agents. J. Med. Chem..

[32] Swietnicki W., Goldeman W., Psurski M., Nasulewicz-Goldeman A., Boguszewska-Czubara A., Drab M., Sycz J., Goszczyński T. M. (2021). Metallacarborane
Derivatives Effective against Pseudomonas Aeruginosa and Yersinia
Enterocolitica. Int. J. Mol. Sci..

[33] Materials C., Borrós S., Viñas C., Teixidor F. (2000). Are Low-Coordinating
Anions of Interest as Doping Agents in Organic Conducting Polymers?. Adv.mater..

[34] Masalles C., Llop J., Viñas C., Teixidor F. (2002). Extraordinary Overoxidation
Resistance Increase in Self-Doped Polypyrroles by Using Non-conventional
Low Charge-Density Anions. Adv. Mater..

[35] Housecroft, C. E. Boron: Metallacarbaboranes. InEncyclopedia of Inorganic and Bioinorganic Chemistry; John Wiley: New York, 2011.

[36] Sivaev I.
B., Bregadze V. I. B. (2018). Carborane
and Metallacarborane Anions for Stabilization
of Transient and Highly Reactive Intermediates. Boron. Organomet. Chem..

[37] Cabrera-González J., Chaari M., Teixidor F., Viñas C., Núñez R. (2020). Blue Emitting Star-Shaped and Octasilsesquioxane-Based
Polyanions Bearing Boron Clusters. Photophysical and Thermal Properties. Molecules.

[38] Rak J., Dejlová B., Lampová H., Kaplánek R., Matějíček P., Cígler P., Král V. (2013). On the Solubility and Lipophilicity
of Metallacarborane
Pharmacophores. Mol. Pharmaceutics.

[39] Viñas C., Tarrés M., González-Cardoso P., Farràs P., Bauduin P., Teixidor F. (2014). Surfactant Behaviour of Metallacarboranes.
A Study Based on the Electrolysis of Water. Dalton Trans..

[40] Gassin P.M., Girard L., Martin-Gassin G., Brusselle D., Jonchere A., Diat O., Viñas C., Teixidor F., Bauduin P. (2015). Surface Activity and Molecular Organization
of Metallacarboranes at the Air – Water Interface Revealed
by Nonlinear Optics. Langmuir.

[41] Brusselle D., Bauduin P., Girard L., Zaulet A., Viñas C., Teixidor F., Ly I., Diat O. (2013). Lyotropic Lamellar
Phase Formed from Monolayered -Shaped Carborane-Cage Amphiphiles. Angew. Chem., Int. Ed..

[42] Fernandez-Alvarez R., Ďordovič V., Uchman M., Matějíček P. (2018). Amphiphiles
without Head-and-Tail Design: Nanostructures Based on the Self-Assembly
of Anionic Boron Cluster Compounds. Langmuir.

[43] Uchman M., Ďordovič V., Tošner Z., Matějíček P. (2015). Classical Amphiphilic Behavior of
Nonclassical Amphiphiles: A Comparison of Metallacarborane Self-Assembly
with SDS Micellization Angewandte. Angew. Chem.
Int. Ed..

[44] Tarrés M., Canetta E., Viñas C., Teixidor F., Harwood A.J. (2014). Imaging
in living cells using ν B–H Raman spectroscopy: Monitoring
COSAN uptake. Chem. Commun..

[45] Teixidor F., Canetta E., Forbes J., Harwood A.J., Tarrés M., Viñas C., Paul E., Azzouni K. (2015). Biological Interaction
of Living Cells with COSAN-Based Synthetic Vesicles. Sci. Rep..

[46] Chaari M., Gaztelumendi N., Cabrera-González J., Peixoto-Moledo P., Viñas C., Xochitiotzi-Flores E., Farfán N., Ben Salah A., Nogués C., Núñez R. (2018). Fluorescent
BODIPY-Anionic Boron Cluster Conjugates as Potential Agents for Cell
Tracking. Bioconjugate Chem..

[47] Ferrer-Ugalde A., Sandoval S., Pulagam K. R., Muñoz-Juan A., Laromaine A., Llop J., Tobias G., Núñez R. (2021). Radiolabeled
Cobaltabis­(Dicarbollide) Anion-Graphene Oxide Nanocomposites for in
Vivo Bioimaging and Boron Delivery. ACS Appl.
Nano Mater..

[48] Ferrer-Ugalde A., Muñoz-Juan A., Laromaine A., Curotto P., Nievas S., Dagrosa M. A., Couto M., Núñez R. (2025). Enhancing
Boron Neutron Capture Therapy (BNCT) with Materials Based on COSAN-Functionalized
Nanoparticles. Pharmaceuticals.

[49] Muñoz-Flores B. M., Cabrera-Gonzalez J., Viñas C., Chavez-Reyes A., Dias H. V. R., Jiménez-Pérez V. M., Núñez R. (2018). Organotin Dyes Bearing Anionic Boron Clusters as Cell-Staining
Fluorescent Probes. Chem. Eur. J.,.

[50] Malaspina D. C., Viñas C., Teixidor F., Faraudo J. (2020). Atomistic Simulations
of COSAN: Amphiphiles without a Head-and-Tail Design Display “Head
and Tail” Surfactant Behavior. Angew.
Chem., Int. Ed..

[51] Chen Y., Barba-Bon A., Grüner B., Winterhalter M., Aksoyoglu M. A., Pangeni S., Ashjari M., Brix K., Salluce G., Folgar-Cameán Y., Montenegro J., Nau W. M. (2023). Metallacarborane Cluster Anions of the Cobalt Bisdicarbollide-Type
as Chaotropic Carriers for Transmembrane and Intracellular Delivery
of Cationic Peptides. J. Am. Chem. Soc..

[52] García-Mendiola T., Bayon-Pizarro V., Zaulet A., Fuentes I., Pariente F., Teixidor F., Viñas C., Lorenzo E. (2016). Metallacarboranes as
Tunable Redox Potential Electrochemical Indicators for Screening of
Gene Mutation. Chem. Sci..

[53] Nuez-Martínez M., Queralt-Martín M., Muñoz-Juan A., Aguilella V. M., Laromaine A., Teixidor F., Viñas C., Pinto C. G., Pinheiro T., Guerreiro J. F., Mendes F., Roma-Rodrigues C., Baptista P. V., Fernandes A. R., Valic S., Marques F. (2022). Boron Clusters
(Ferrabisdicarbollides)
Shaping the Future as Radiosensitizers for Multimodal (Chemo/Radio/PBFR)
Therapy of Glioblastoma. J. Mater. Chem. B.

[54] Zhao L., Liu X., Zhang L., Qiu G., Astruc D., Gu H. (2017). Metallomacromolecules
Containing Cobalt Sandwich Complexes: Synthesis and Functional Materials
Properties. Coord. Chem. Rev..

[55] Axtell J. C., Saleh L. M. A., Qian E. A., Wixtrom A. I., Spokoyny A. M. (2018). Synthesis
and Applications of Perfunctionalized Boron Clusters. Inorg. Chem..

[56] Assaf K. I., Ural M. S., Pan F., Georgiev T., Simova S., Rissanen K., Gabel D., Nau W. M. (2015). Water Structure
Recovery in Chaotropic Anion Recognition: High-Affinity Binding of
Dodecaborate Clusters to Γ-Cyclodextrin. Angew. Chem., Int. Ed..

[57] Barba-Bon A., Salluce G., Lostalé-Seijo I., Assaf K. I., Hennig A., Montenegro J., Nau W. M. (2022). Boron Clusters as
Broadband Membrane Carriers. Nature.

[58] Shi C., Sun H., Tang X., Lv W., Yan H., Zhao Q., Wang J., Huang W. (2013). Variable Photophysical Properties
of Phosphorescent Iridium­(III) Complexes Triggered by Closo- and Nido-Carborane
Substitution. Angew. Chem., Int. Ed..

[59] Sun Z., Zong J., Ren H., Lu C., Tu D., Poater J., Solà M., Shi Z., Yan H. (2024). Couple-Close
Construction of Non-Classical Boron Cluster-Phosphonium Conjugates. Nat. Commun..

[60] Bellomo C., Zanetti D., Cardano F., Sinha S., Chaari M., Fin A., Maranzana A., Núñez R., Blangetti M., Prandi C. (2021). Red Light-Emitting
Carborane-BODIPY Dyes: Synthesis
and Properties of Visible-Light Tuned Fluorophores with Enhanced Boron
Content. Dyes Pigm.

[61] Labra-Vázquez P., Flores-Cruz R., Galindo-Hernández A., Cabrera-González J., Guzmán-Cedillo C., Jiménez-Sánchez A., Lacroix P. G., Santillan R., Farfán N., Núñez R. (2020). Tuning the Cell Uptake and Subcellular Distribution
in BODIPY–Carboranyl Dyads: An Experimental and Theoretical
Study. Chem.– Eur. J..

[62] Bellomo C., Chaari M., Cabrera-González J., Blangetti M., Lombardi C., Deagostino A., Viñas C., Gaztelumendi N., Nogués C., Núñez R. (2018). Carborane-BODIPY Dyads: New Photoluminescent
Materials through an
Efficient Heck Coupling. Chem.– Eur.
J..

[63] Ordóñez-Hernández J., Planas J. G., Núñez R. (2024). Carborane-Based BODIPY Dyes: Synthesis,
Structural Analysis, Photophysics and Applications. Front. Chem..

[64] Mahanta C. S., Hansdah S., Khuntia K., Jena B. B., Swain B. R., Acharya S., Dash B. P., Debata P. R., Satapathy R. (2024). Novel Carboranyl-BODIPY
Conjugates: Design, Synthesis and Anti-Cancer Activity. RSC Adv..

[65] Sivaev I. B., Kulikova N. Y., Nizhnik E. A., Vichuzhanin M. V., Starikova Z. A., Semioshkin A. A., Bregadze V. I. (2008). Practical Synthesis
of 1,4-Dioxane Derivative of the Closo-Dodecaborate Anion and Its
Ring Opening with Acetylenic Alkoxides. J. Organomet.
Chem..

[66] Teixidor F., Pedrajas J., Rojo I., Viñas C., Kivekäs R., Sillanpää R., Sivaev I., Bregadze V., Sjöberg S. (2003). Chameleonic
Capacity of [3,3′-Co­(1,2-C2B9H
11)­2]- in Coordination. Generation of the Highly Uncommon S­(Thioether)-Na
Bond. Organometallics.

[67] Cioran A. M., Teixidor F., Viñas C. (2015). The Effect
of a Paramagnetic Metal
Ion within a Molecule: Comparison of the Structurally Identical Paramagnetic
[3,3-Fe­(1,2-C2B9H11)­2]- with the Diamagnetic [3,3-Co­(1,2-C2B9H11)­2]-
Sandwich Complexes. Dalton Trans.

[68] Caruso E., Banfi S., Barbieri P., Leva B., Orlandi V. T. (2012). Synthesis
and Antibacterial Activity of Novel Cationic BODIPY Photosensitizers. J. Photochem. Photobiol., B.

[69] Solov’ev K. N., Borisevich E. A. (2005). Intramolecular
Heavy-Atom Effect in the Photophysics
of Organic Molecules. Physics-Usp..

[70] Klan, P. ; Wirz, J. Photochemistry of organic compounds: From concepts to practice; John Wiley, 2009.

[71] Chen K., Dong Y., Zhao X., Imran M., Tang G., Zhao J., Liu Q. (2019). Bodipy Derivatives
as Triplet Photosensitizers
and the Related Intersystem Crossing Mechanisms. Front. Chem..

[72] Ly J. T., Presley K. F., Cooper T. M., Baldwin L. A., Dalton M. J., Grusenmeyer T. A. (2021). Impact of Iodine Loading and Substitution
Position
on Intersystem Crossing Efficiency in a Series of Ten Methylated-:
Meso -Phenyl-BODIPY Dyes. Phys. Chem. Chem.
Phys..

[73] Doležel J., Poryvai A., Slanina T., Filgas J., Slavíček P. (2024). Spin-Vibronic
Coupling Controls the Intersystem Crossing of Iodine-Substituted BODIPY
Triplet Chromophores. Chem.– Eur. J..

[74] Spada R. M., Cepeda-Plaza M., Gómez M. L., Günther G., Jaque P., Pizarro N., Palacios R. E., Vega A. (2015). Clean Singlet
Oxygen Production by a ReI Complex Embedded in a Flexible Self-Standing
Polymeric Silsesquioxane Film. J. Phys. Chem.
C.

[75] Albiter E., Alfaro S., Valenzuela M. A. (2015). Photosensitized
Oxidation of 9,10-Dimethylanthracene
with Singlet Oxygen by Using a Safranin O/Silica Composite under Visible
Light. Photochem. Photobiol. Sci..

[76] Entradas T., Waldron S., Volk M. (2020). The Detection
Sensitivity of Commonly
Used Singlet Oxygen Probes in Aqueous Environments. J. Photochem. Photobiol., B.

[77] Ji Y., Li J., Chen C., Piao C., Zhou X., Yoon J. (2024). Wash-Free
Bacterial Gram-Typing and Photodynamic Inactivation with Long-Chain-Tailed
BODIPY Derivatives. Biomater. Res..

[78] Delcanale P., Abbruzzetti S., Viappiani C. (2022). Photodynamic Treatment of Pathogens. Rivista Del Nuovo Cimento.

[79] Xu J., Huang H., Wang K., Zhu J., Zhao J., Zhao Y., Yue J., Ying C., Tao W., Tong Q., Quan L., Xie J. (2024). Design and Synthesis
of BODIPY and Its Application in Inhibiting Intestinal Flora. ACS Omega.

[80] Carpenter B. L., Situ X., Scholle F., Bartelmess J., Weare W. W., Ghiladi R. A. (2015). Antiviral Antifungal
and Antibacterial
Activities of a BODIPY-Based Photosensitizer. Molecules.

[81] Banfi S., Caruso E., Buccafurni L., Battini V., Zazzaron S., Barbieri P., Orlandi V. (2006). Antibacterial
Activity of Tetraaryl-Porphyrin
Photosensitizers: An in Vitro Study on Gram Negative and Gram Positive
Bacteria. J. Photochem. Photobiol., B.

[82] Bierge P., Gómez-Sánchez I., García de la Mària C., Sánchez-Osuna M., Capilla S., Casabella A., Espasa M., Novais C., Miró J. M., Pich O. Q. (2025). Is Ampicillin plus Cephalosporins a Therapeutic Option
for Ampicillin-Susceptible Enterococcus Faecium?. J. Antimicrob. Chemother.

[83] Gómez A. C., Lyons T., Mamat U., Yero D., Bravo M., Daura X., Elshafee O., Brunke S., Gahan C. G. M., O’Driscoll M. (2022). Synthesis and Evaluation
of Novel Furanones as Biofilm Inhibitors in Opportunistic Human Pathogens. Eur. J. Med. Chem..

