# Supplementary material for: Anionic Boron-Cluster BODIPY Conjugates As Promising Photosensitizers for Targeted Antimicrobial Photodynamic Therapy
Source: ACS Omega. 2025 Sep 16;10(38):44693–705. doi: 10.1021/acsomega.5c07634 (PMC12489852; doi:10.1021/acsomega.5c07634)
Supplement: Supplementary file 1 [file ao5c07634_si_001.pdf]

Anionic Boron-Cluster BODIPY Conjugates as Promising Photosensitizers for Targeted Antimicrobial Photodynamic Therapy

Javier Ordóñez-Hernández,[a] Andromeda-Celeste Gómez,[b,c] Jordi Hernando,[d] Marina Quevedo,[b,c] Juan Camilo Ortíz,[b,c] Daniel Yero,[b,c] Isidre Gibert,[b,c] Rosario Núñez[a*]

[a]Inorganic Materials and Catalysis Laboratory (LMI), Institut de Ciència de Materials de Barcelona (ICMAB-CSIC), Campus de la UAB, 08193 Bellaterra (Cerdanyola del Vallès), Barcelona, Spain

[b]Institut de Biotecnologia i de Biomedicina, Universitat Autònoma de Barcelona (UAB), 08193 Bellaterra (Cerdanyola del Vallès), Barcelona, Spain.

[c]Departament de Genètica i de Microbiologia, Universitat Autònoma de Barcelona (UAB), 08193 Bellaterra (Cerdanyola del Vallès), Barcelona, Spain

[d]Departament de Química. Universitat Autònoma de Barcelona, (UAB), 08193 Bellaterra (Cerdanyola del Vallès), Barcelona, Spain.

CONTENTS

1. Nuclear Magnetic Resonance Spectra

Figure S1. ^{1}H NMR spectrum of **BDP** in $CO(CD_3)_2$ at 300 MHz. 1

Figure S2. ^{13}C NMR spectrum of **BDP** in $CO(CD_3)_2$ at 75 MHz. 1

Figure S3. ^{19}F NMR and ^{11}B NMR spectra at 282.4 MHz and 96.3 MHz, of **BDP** in $CO(CD_3)_2$ 2

Figure S4. ^{1}H NMR spectrum of **BDP-I₂** in $CDCl_3$ at 300 MHz 2

Figure S5. ^{13}C NMR spectrum of **BDP-I₂** in $CDCl_3$ at 75 MHz 3

Figure S6. ^{19}F NMR and ^{11}B NMR spectra at 282.4 MHz and 96.3 MHz of **BDP** in $CDCl_3$ 3

Figure S7. ^{1}H NMR spectrum of **BDP-FES** in $CO(CD_3)_2$ at 300 MHz 4

Figure S8. ^{13}C NMR spectrum of **BDP-FES** in $CO(CD_3)_2$ at 75 MHz 4

Figure S9. ^{19}F NMR spectrum of **BDP-FES** in $CO(CD_3)_2$ at 282.4 MHz 5

Figure S10. $^{11}B\{^{1}H\}$ NMR spectrum of **BDP-FES** in $CO(CD_3)_2$ at 96.3 MHz 5

Figure S11. ^{1}H NMR spectrum of **BDP-COS** in $CO(CD_3)_2$ at 300 MHz 6

Figure S12. ^{13}C NMR spectrum of **BDP-COS** in $CO(CD_3)_2$ at 75 MHz 6

Figure S13. ^{19}F NMR spectrum of **BDP-COS** in $CO(CD_3)_2$ at 282.4 MHz 7

Figure S14. $^{11}B\{^{1}H\}$ NMR spectrum of **BDP-COS** in $CO(CD_3)_2$ at 96.3 MHz 7

Figure S15. ^{1}H NMR spectrum of **BDP-B$_{12}$** in $CO(CD_3)_2$ at 300 MHz 8

Figure S16. ^{13}C NMR spectrum of **BDP-B$_{12}$** in $CO(CD_3)_2$ at 75 MHz 8

Figure S17. ^{19}F NMR spectrum of **BDP-B$_{12}$** in $CO(CD_3)_2$ at 282.4 MHz 9

Figure S18. $^{11}B\{^{1}H\}$ NMR spectrum of **BDP-B$_{12}$** in $CO(CD_3)_2$ at 96.3 MHz 9

2. Cyclic Voltammograms

Figure S19. Cyclic voltammograms of **BDP-I$_2$**, **BDP-FES**, **BDP-COS** and **BDP-B$_{12}$** 10

3. Measurements of Singlet Oxygen Production

Figure S20. Variation of the absorption spectra of a mixture of **BDP** + DMA 11

Figure S21. Variation of the absorption spectra of a mixture of **BDP-I$_2$** + DMA 11

Figure S22. Variation of the absorption spectra of a mixture of **BDP-FES** + DMA 12

Figure S23. Variation of the absorption spectra of a mixture of **BDP-COS** + DMA 12

Figure S24. Variation of the absorption spectra of a mixture of **Rose Bengal** + DMA 13

Figure S25. Variation of the absorption spectra of DMA 13

1. Nuclear Magnetic Resonance Spectra

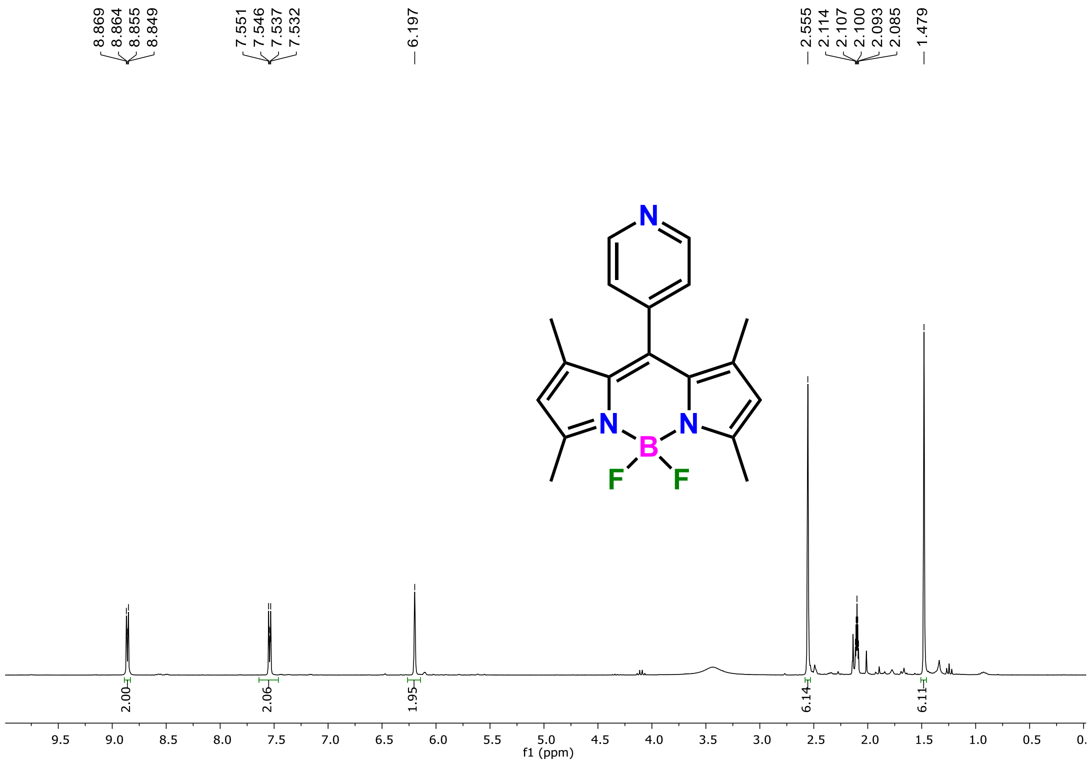

Figure S1. ^1H NMR spectrum of **BDP** in $CO(CD_3)_2$ at 300 MHz.

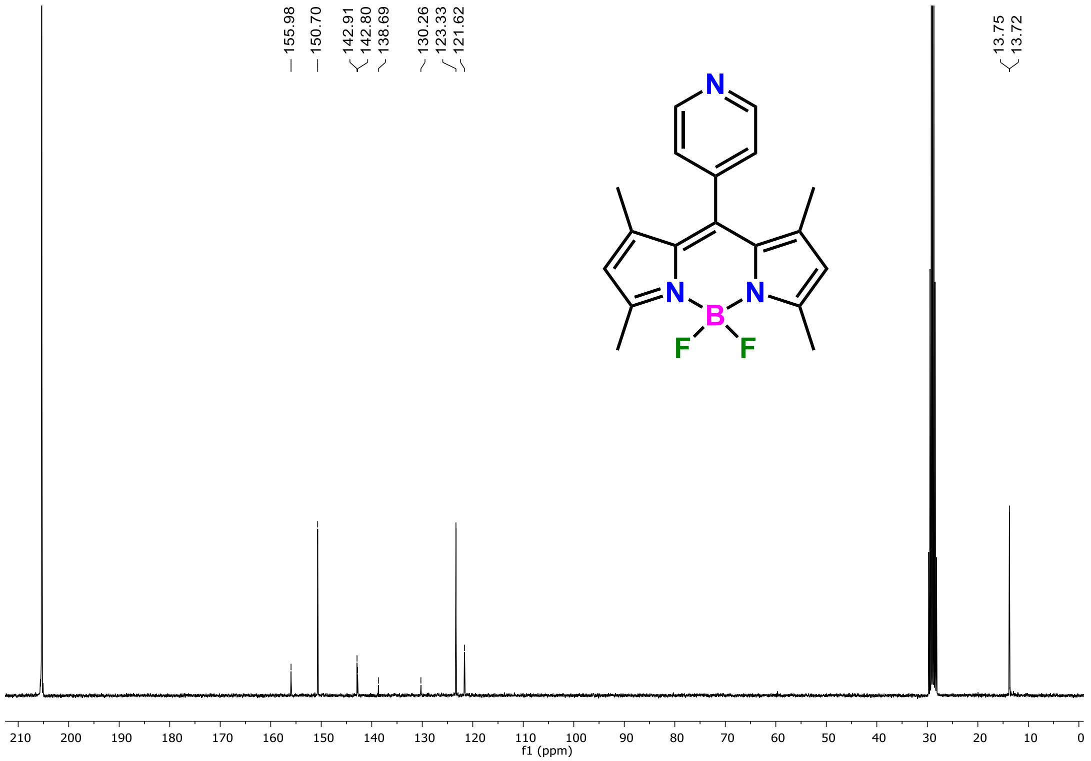

Figure S2. ^{13}C NMR spectrum of **BDP** in $CO(CD_3)_2$ at 75 MHz.

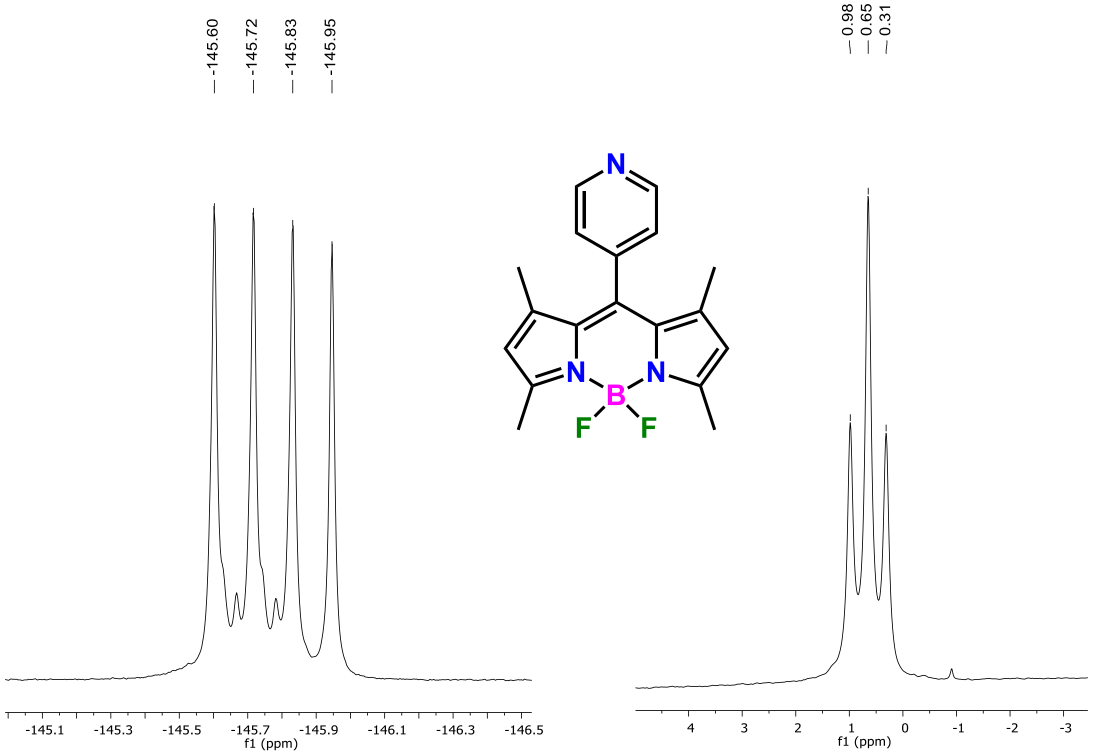

Figure S3. ^{19}F NMR (left) and ^{11}B NMR (right) spectra at 282.4 MHz and 96.3 MHz, respectively of **BDP** in $CO(CD_3)_2$

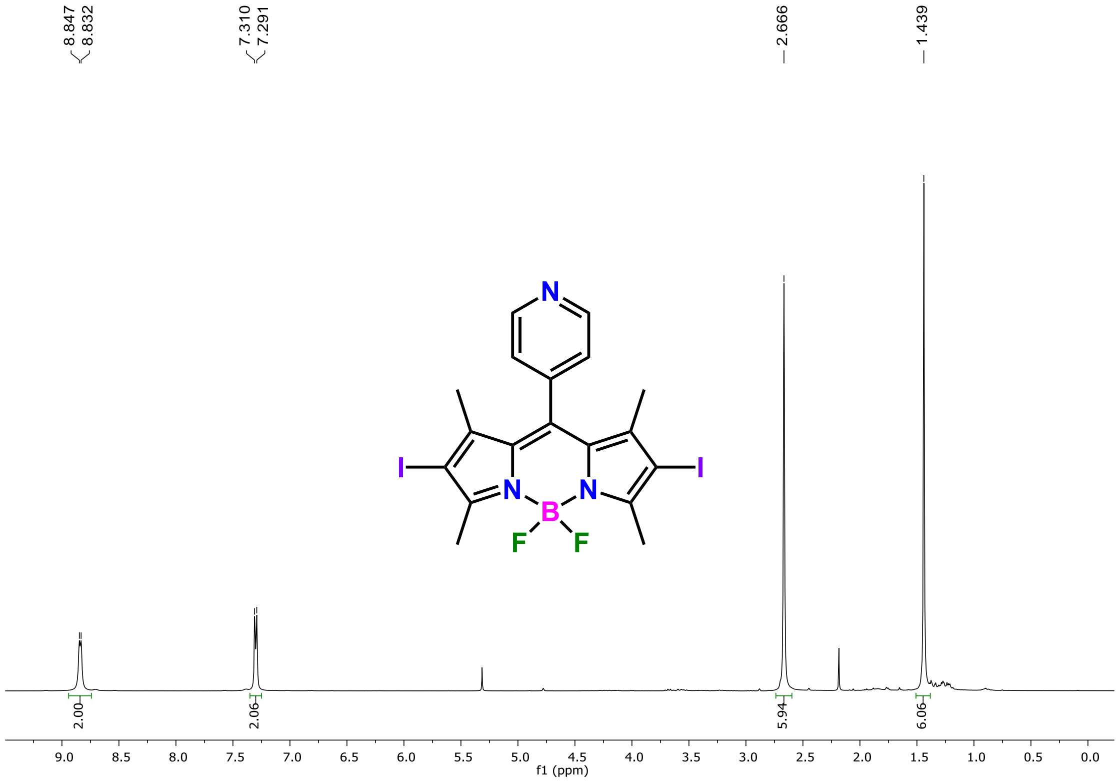

Figure S4. ^{1}H NMR spectrum of **BDP-I₂** in $CDCl_3$ at 300 MHz.

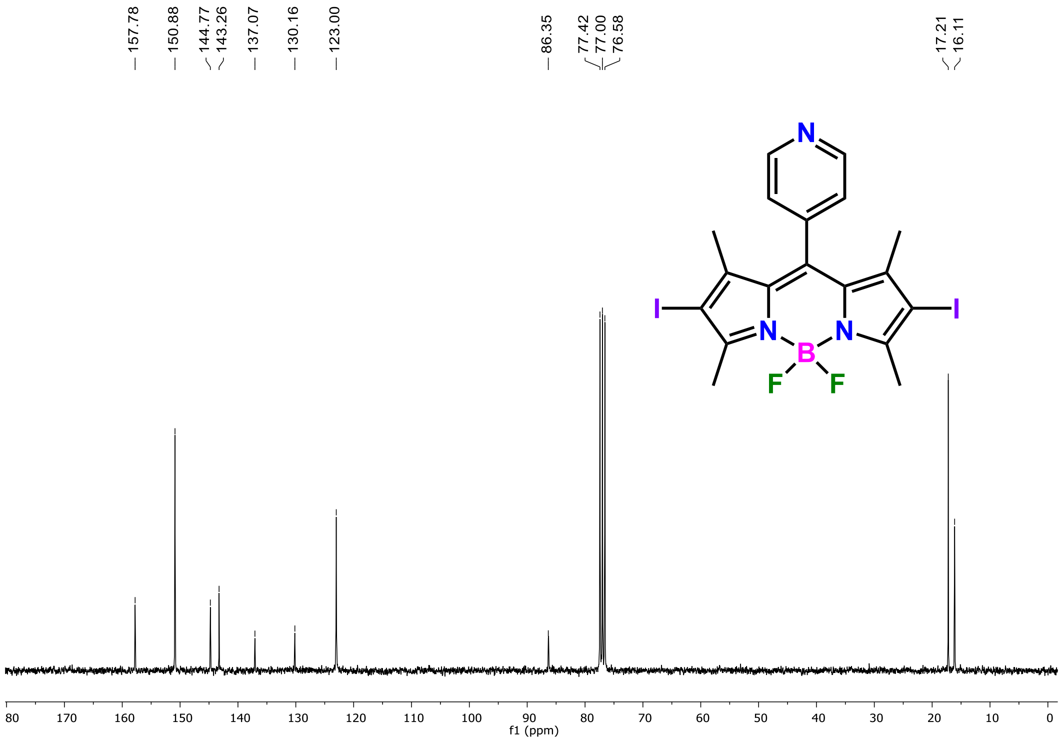

Figure S5. ^{13}C NMR spectrum of **BDP-I$_2$** in $CDCl_3$ at 75 MHz.

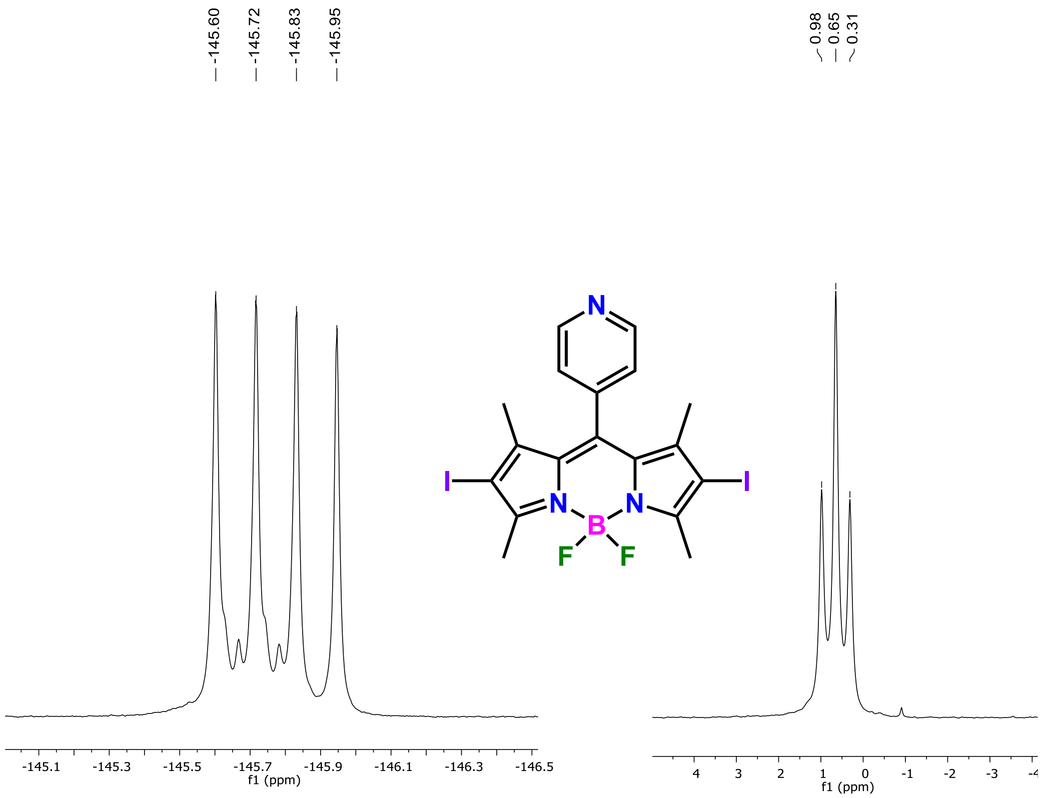

Figure S6. ^{19}F NMR (left) and ^{11}B NMR (right) spectra at 282.4 MHz and 96.3 MHz, respectively, of **BDP-I$_2$** in $CDCl_3$

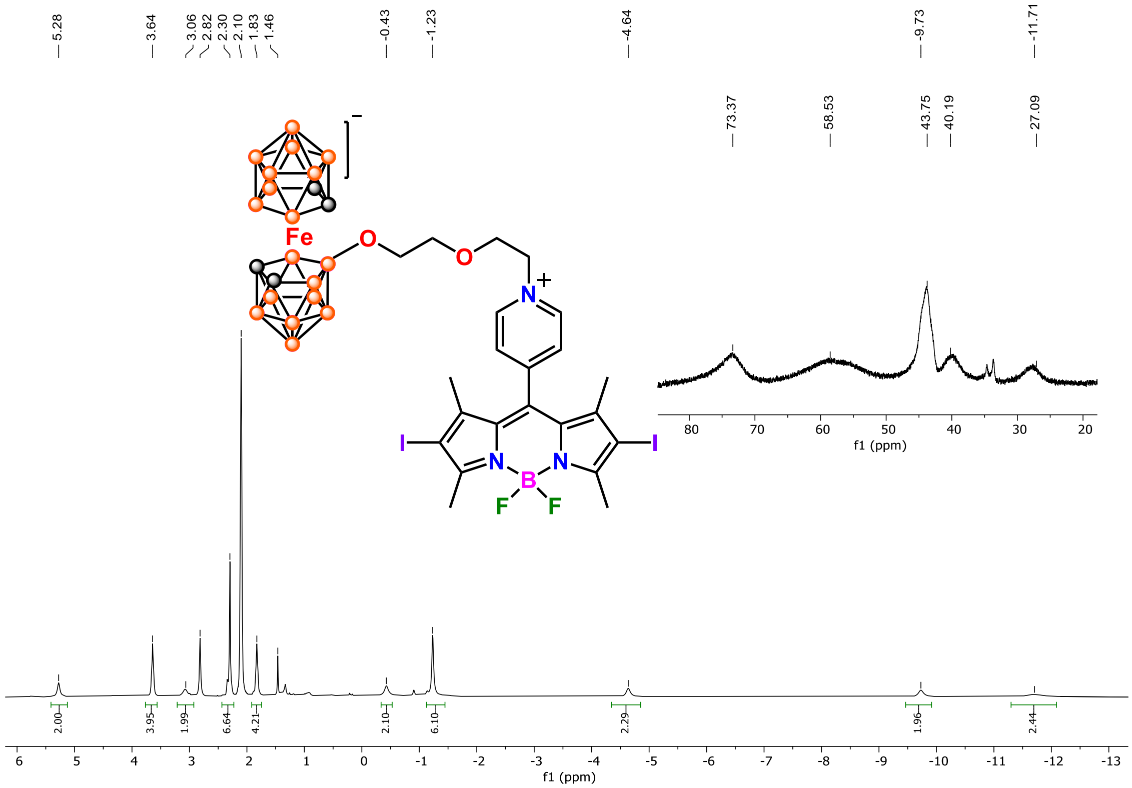

Figure S7. ^{1}H NMR spectrum of **BDP-FES** in $CO(CD_3)_2$ at 300 MHz.

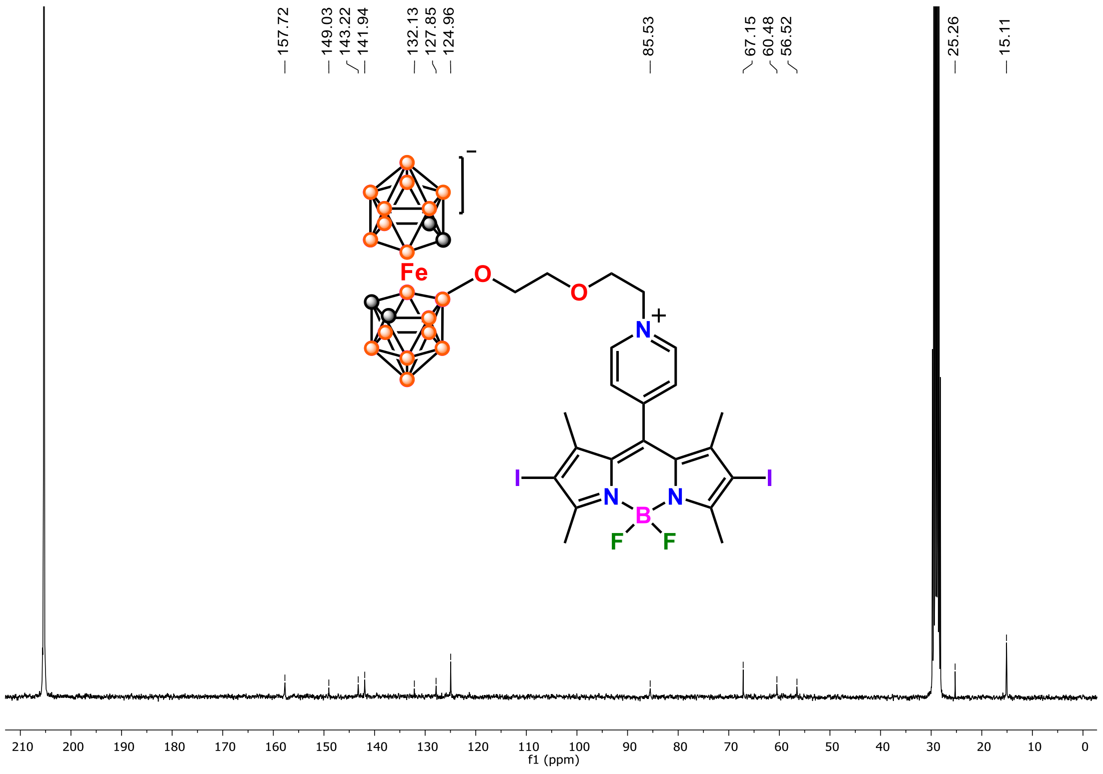

Figure S8. ^{13}C NMR spectrum of **BDP-FES** in in $CO(CD_3)_2$ at 75 MHz.

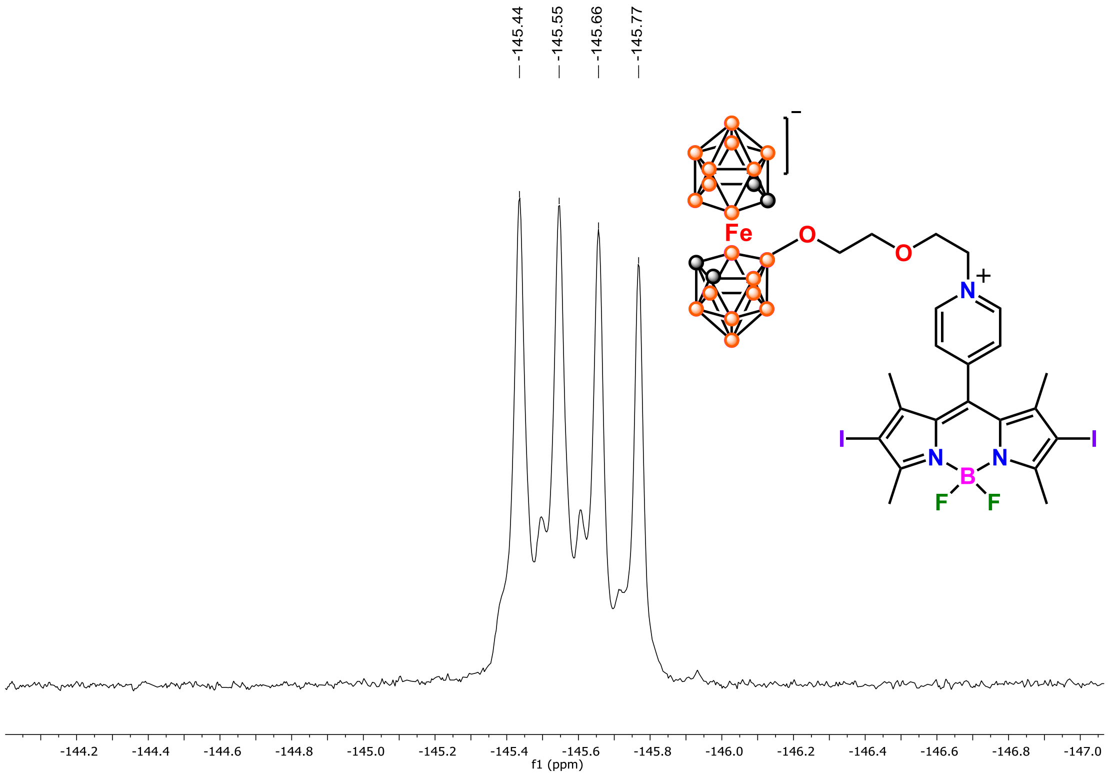

Figure S9. ^{19}F NMR spectrum of **BDP-FES** in $CO(CD_3)_2$ at 282.4 MHz.

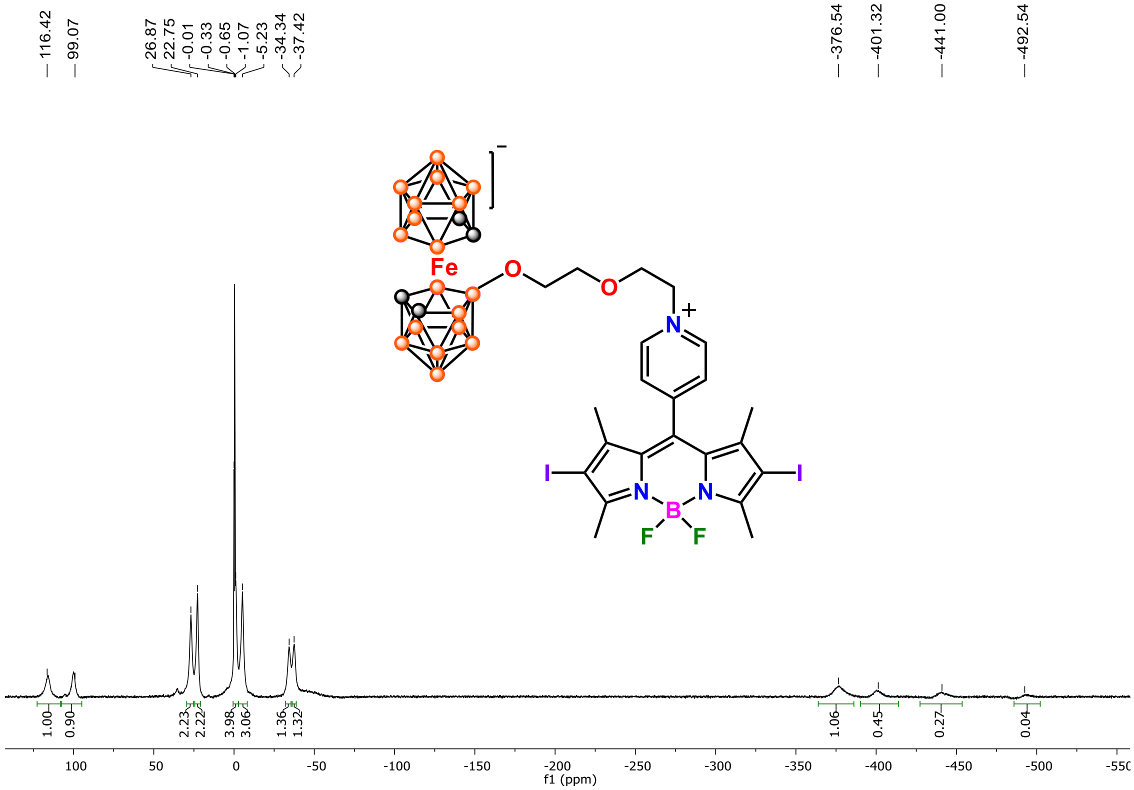

Figure S10. ^{11}B{^{1}H} NMR spectrum of **BDP-FES** in $CO(CD_3)_2$ at 96.3 MHz.

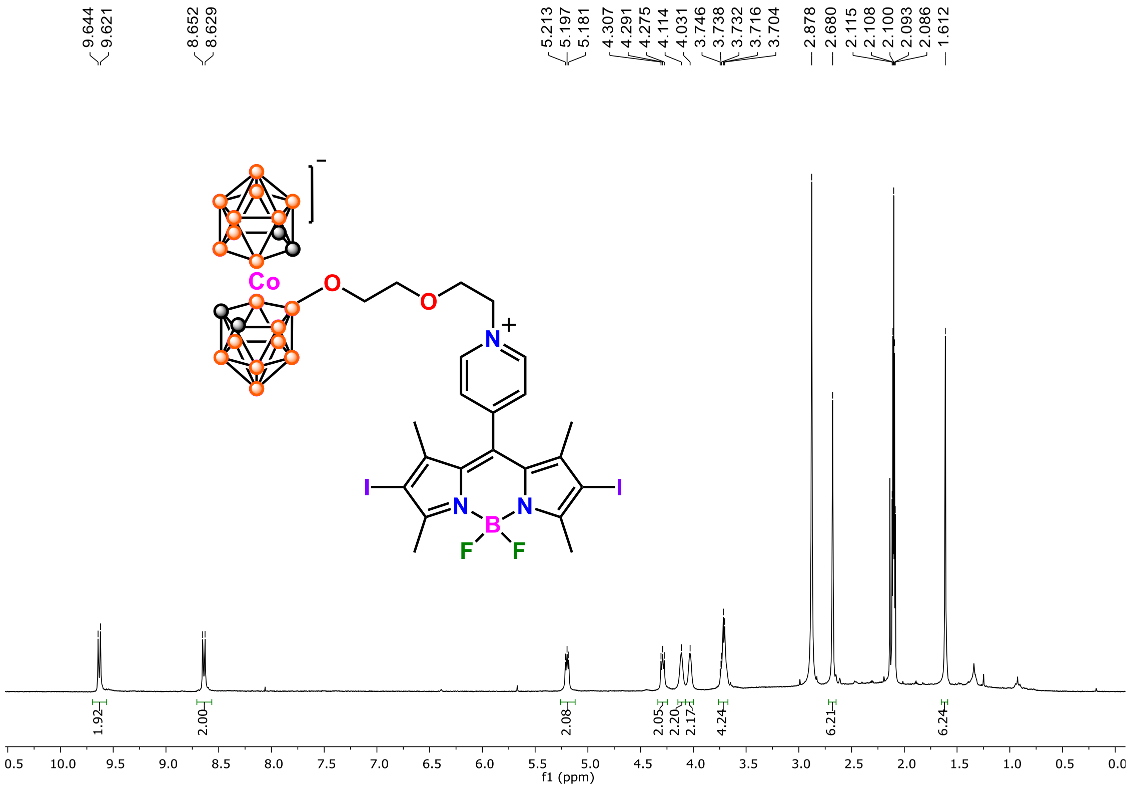

Figure S11. ^{1}H NMR spectrum of **BDP-COS** in $CO(CD_3)_2$ at 300 MHz.

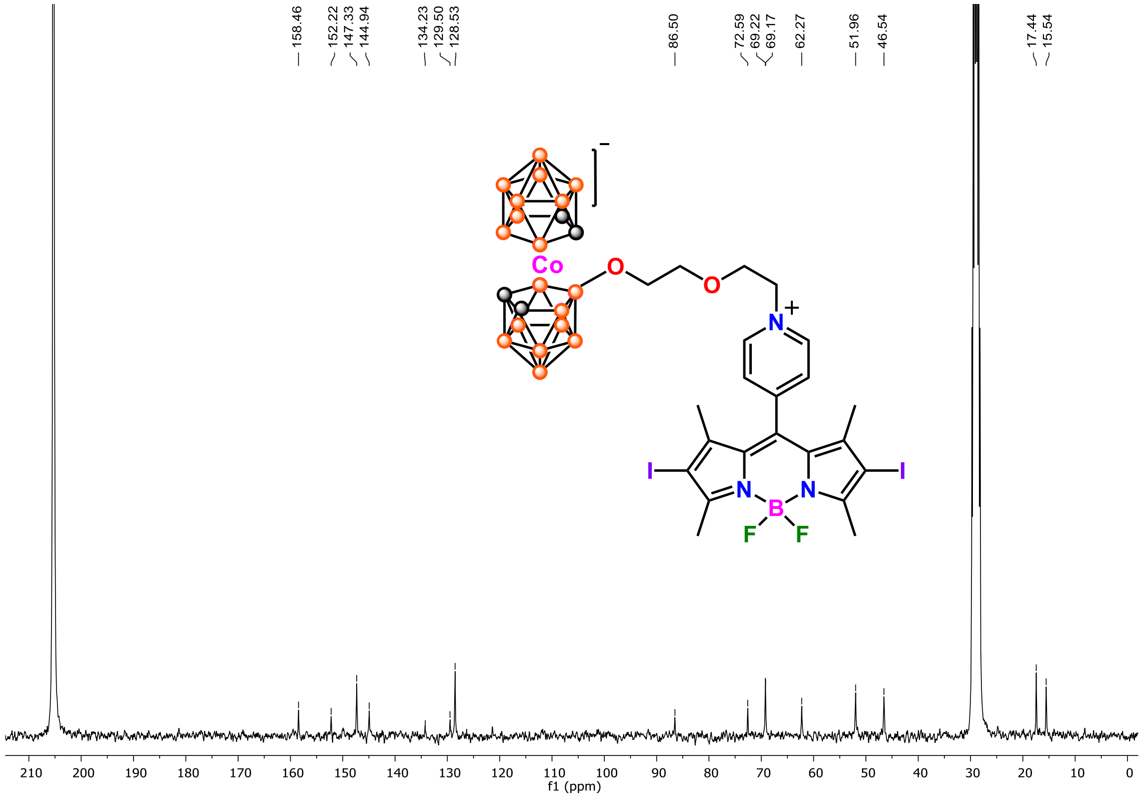

Figure S12. ^{13}C NMR spectrum of **BDP-COS** in $CO(CD_3)_2$ at 75 MHz.

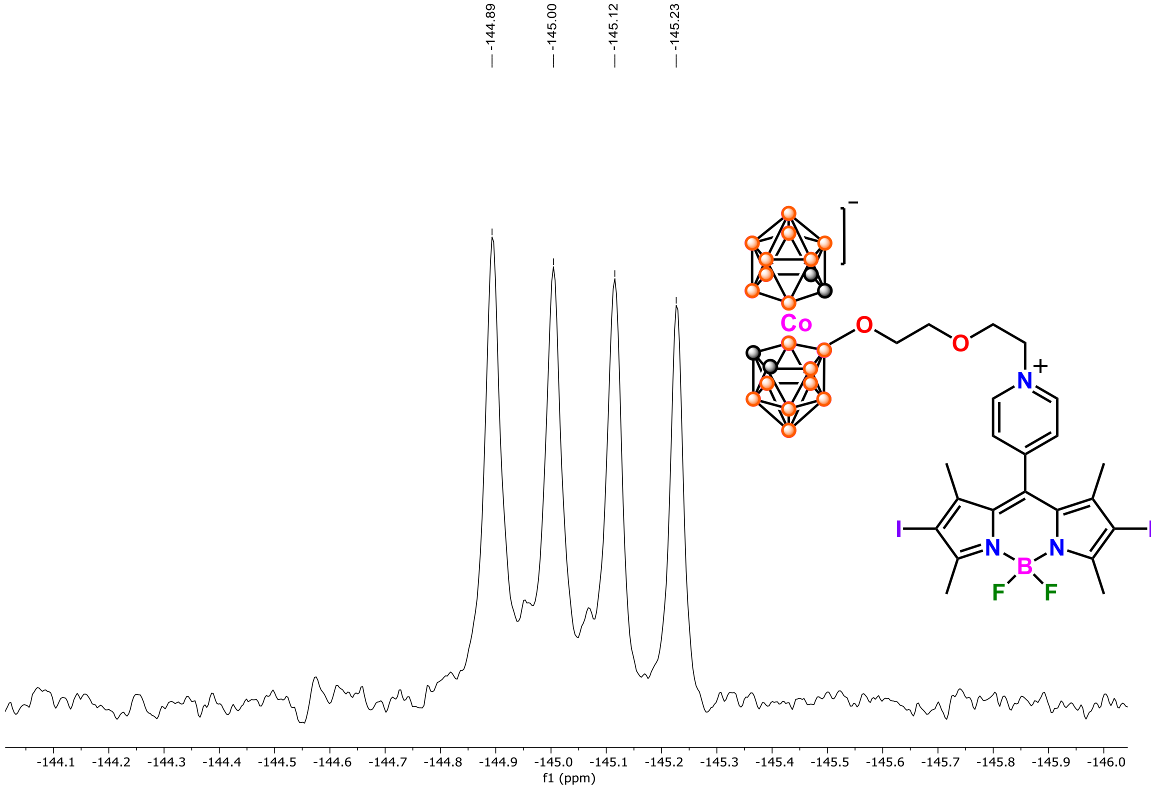

Figure S13. ^{19}F NMR spectrum of **BDP-COS** in $CO(CD_3)_2$ at 282.4 MHz

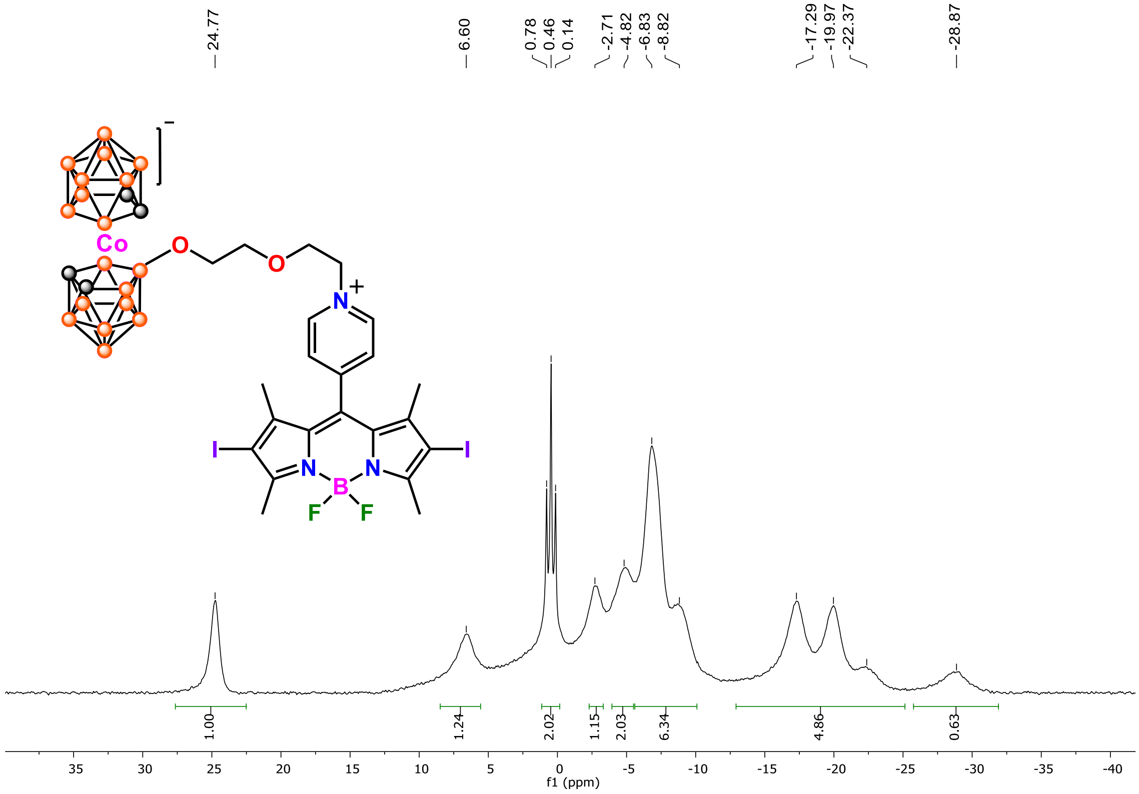

Figure S14. $^{11}B\{^1H\}$ NMR spectrum of **BDP-COS** in $CO(CD_3)_2$ at 96.3 MHz

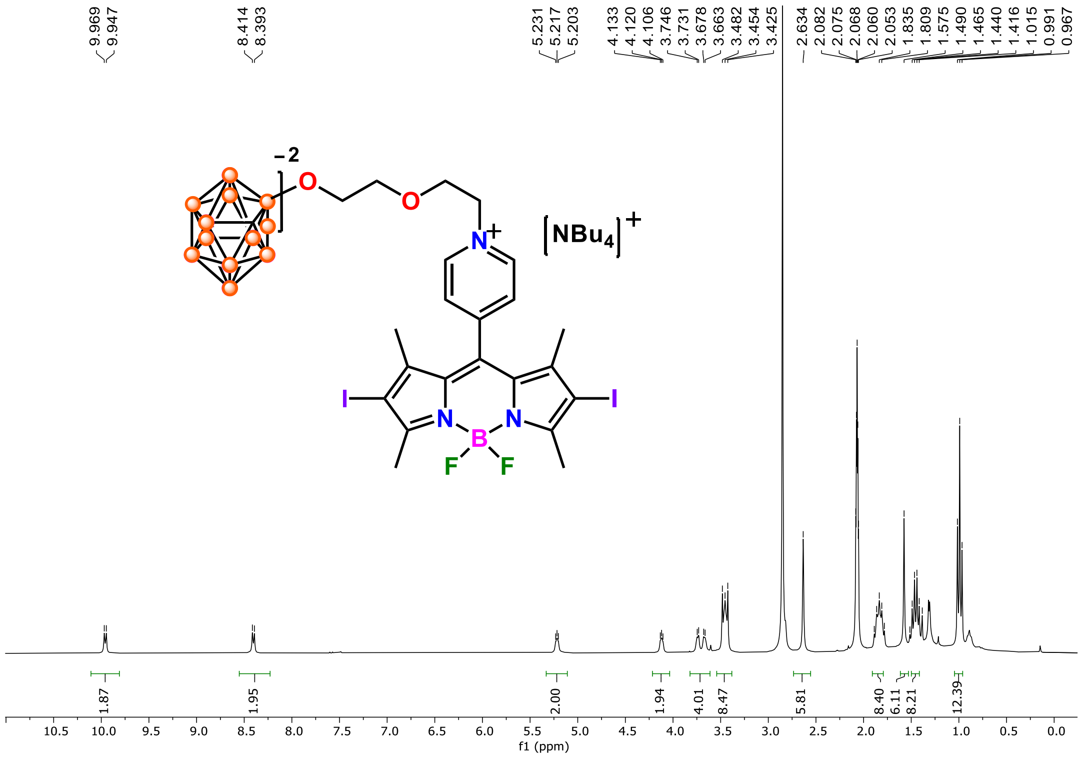

Figure S15. ^{1}H NMR spectrum of **BDP-B12** in $CO(CD_3)_2$ at 300 MHz

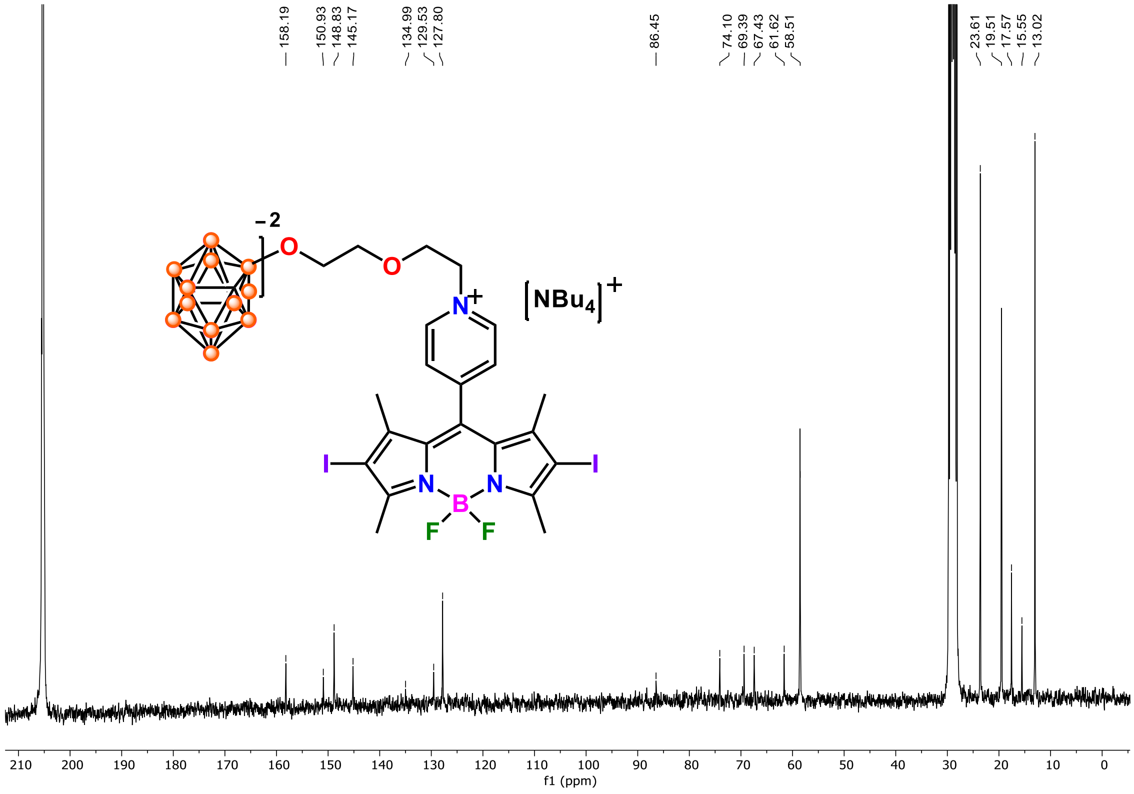

Figure S16. ^{13}C NMR spectrum of **BDP-B12** in $CO(CD_3)_2$ at 75 MHz

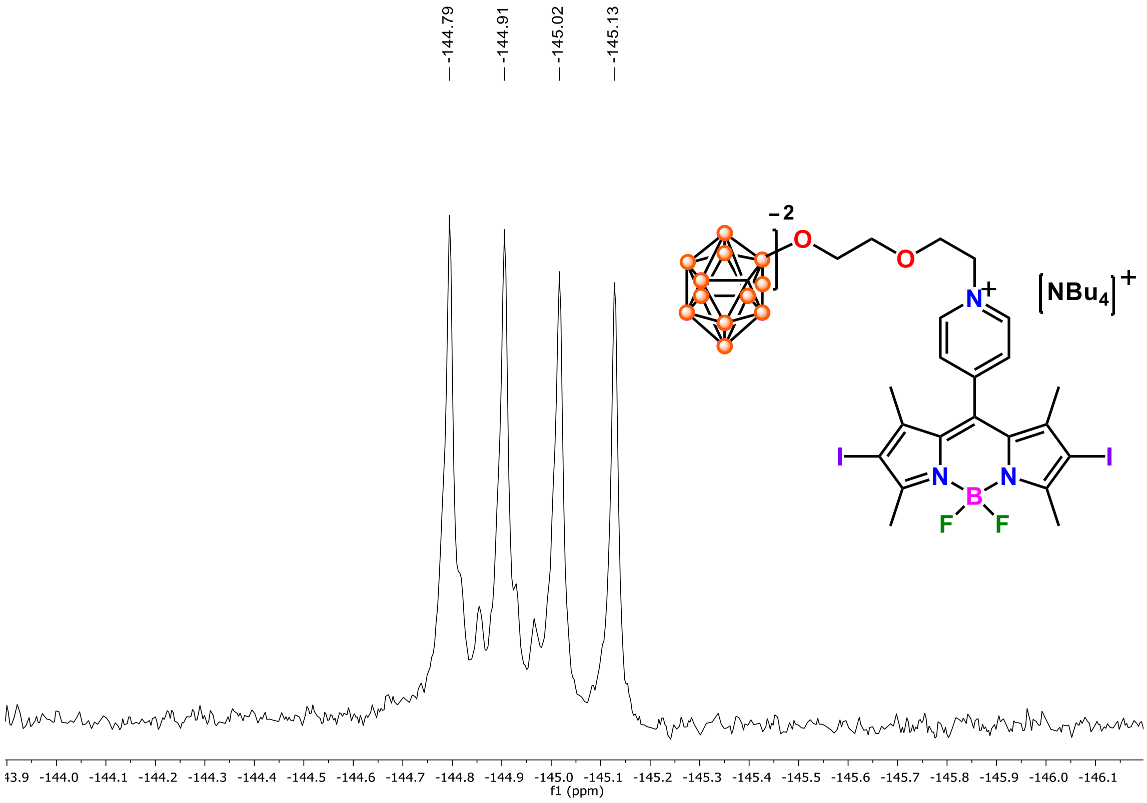

Figure S17. ^{19}F NMR spectrum of **BDP-B$_{12}$** in $CO(CD_3)_2$ at 282.4 MHz

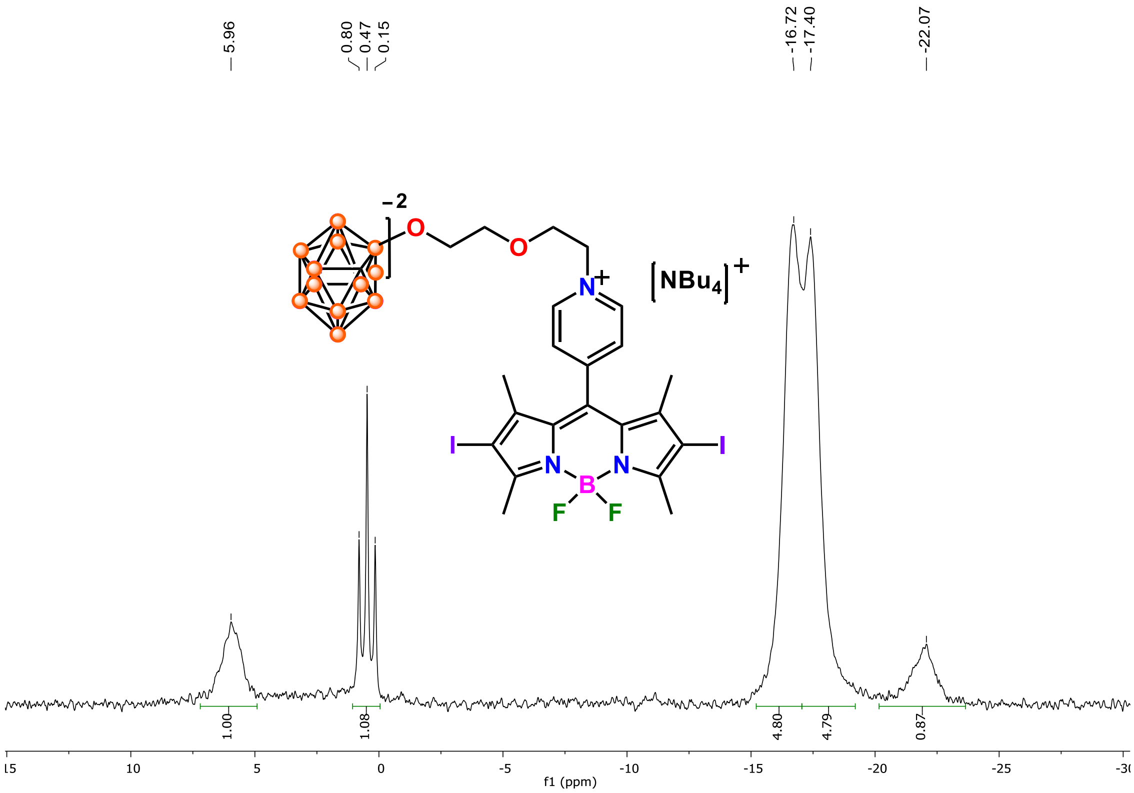

Figure S18. ^{11}B{^{1}H} NMR spectrum of **BDP-B$_{12}$** in $CO(CD_3)_2$ at 96.3 MHz.

2. Cyclic Voltammograms

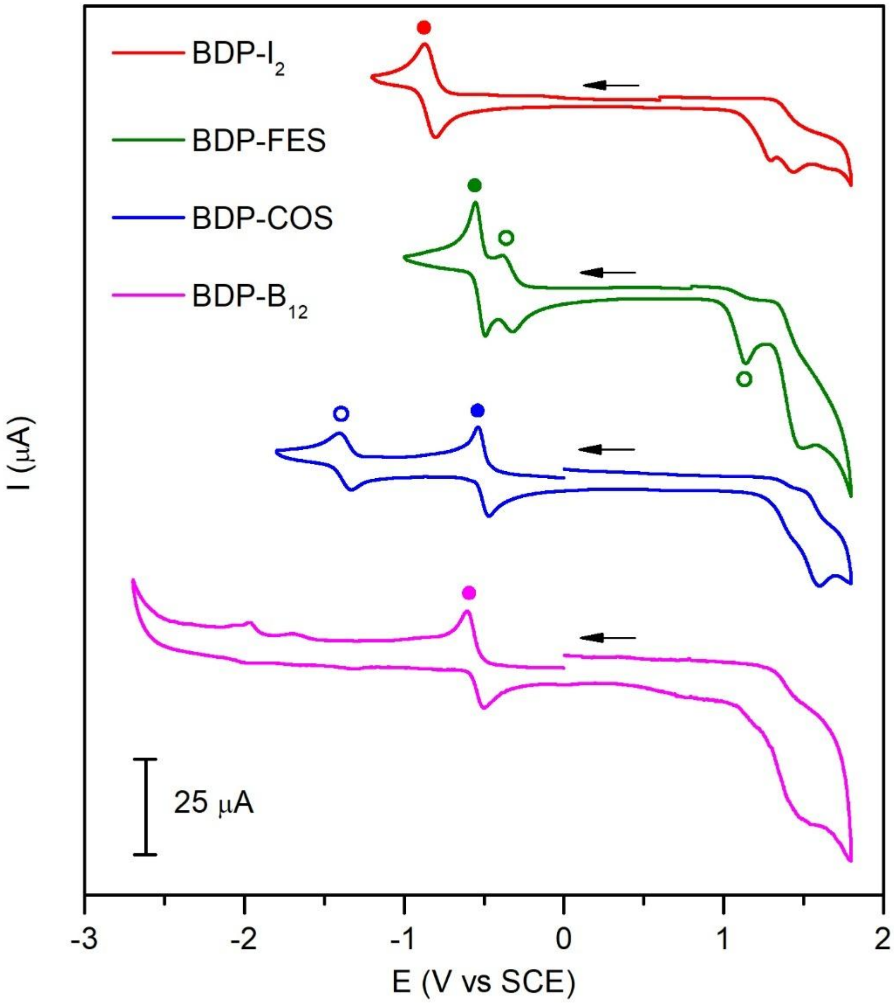

Figure S19. Cyclic voltammograms of **BDP-I2**, **BDP-FES**, **BDP-COS** and **BDP-B12** (c = 0.001 M) in acetonitrile (+0.1 M $TBAPF_6$; scan rate = 0.1 V s^{-1}). The arrows indicate the direction of the potential scan. Full circles are used to mark the reversible reduction wave of the BODIPY chromophore of these compounds, which is slightly more favored for **BDP-FES** (E^0_{red} = -0.522 V (vs SCE)), **BDP-COS** (E^0_{red} = -0.504 V (vs SCE)), and **BDP-B12** (E^0_{red} = -0.552 V (vs SCE)) bearing a cationic *meso*-4-pyridinium substituent relative to **BDP-I2** (E^0_{red} = -0.837 V (vs SCE)). In the cyclic voltammograms of **BDP-FES** and **BDP-COS** additional reversible reduction waves were measured (shown as open circles), which can be attributed to their metallacarborane units by comparison with previous reports:[1] E^0_{red} = -0.352 V (vs SCE) for **BDP-FES**, and E^0_{red} = -1.368 V (vs SCE) for **BDP-COS**. For **BDP-FES**, an additional irreversible oxidation wave was registered and denoted with an open circle ($E_{p,ox}$ = +1.139 V (vs SCE)), which also be assigned to its FESAN cluster.

3. Measurements of Singlet Oxygen Production

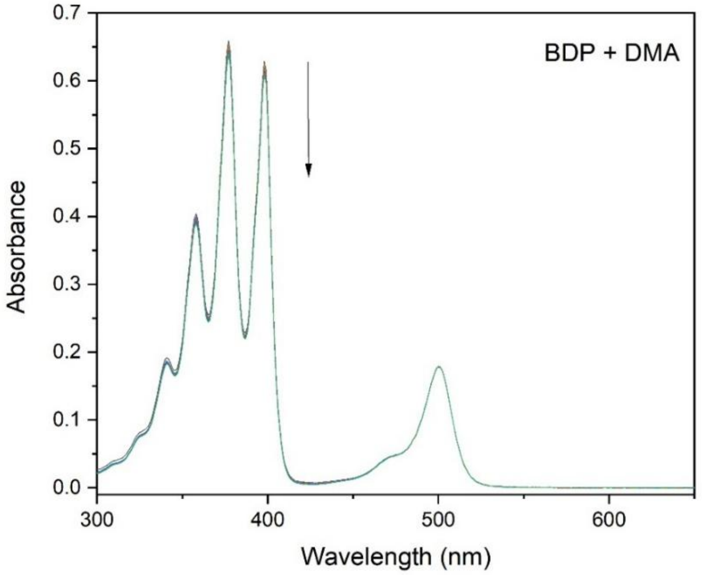

Figure S20. Variation of the absorption spectrum of a mixture of **BDP** (c = 2.7 10^{-6} M) + DMA (c = 6.0 × 10^{-5} M) in acetonitrile upon irradiation with a white light LED through a 435LP filter (power = 15 mW cm^{-2}, λ_{exc} > 435 nm, irradiation from 0 to 600 s).

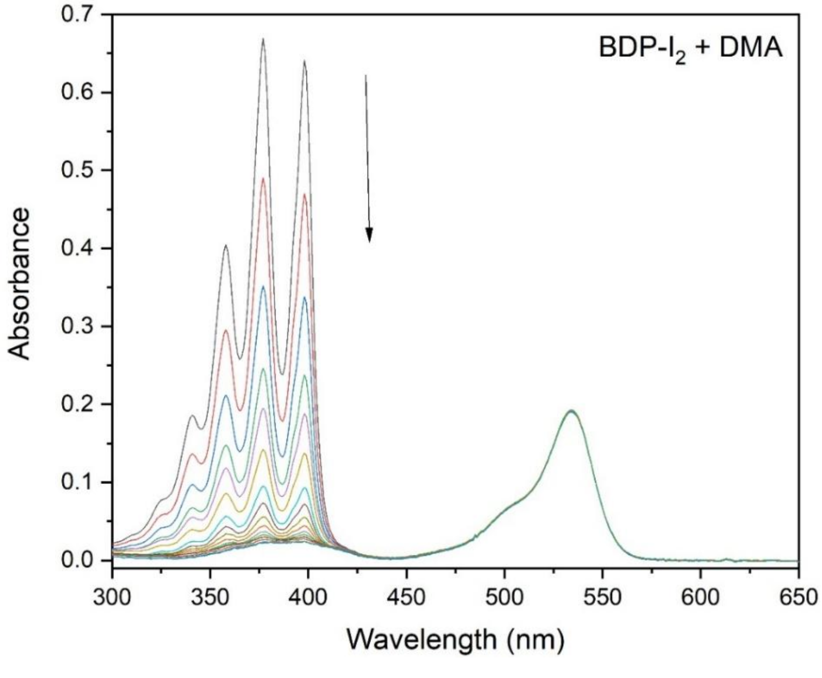

Figure S21. Variation of the absorption spectrum of a mixture of **BDP-I$_2$** (c = 2.7 10^{-6} M) + DMA (c = 6.0 × 10^{-5} M) in acetonitrile upon irradiation with a white light LED through a 435LP filter (power = 15 mW cm^{-2}, λ_{exc} > 435 nm, irradiation from 0 to 480 s).

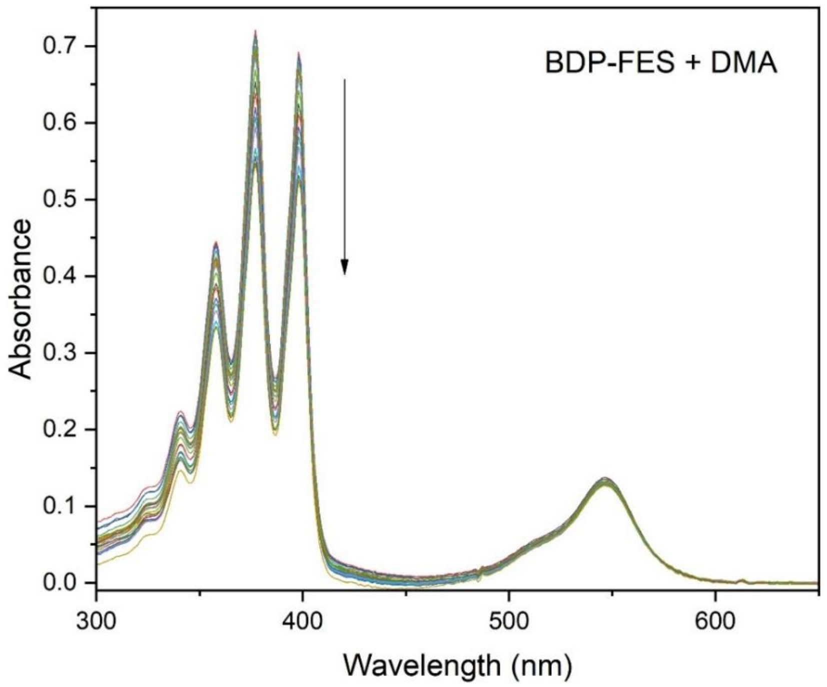

Figure S22. Variation of the absorption spectrum of a mixture of **BDP-FES** (c = 2.7 10^{-6} M) + DMA (c = 6.0 × 10^{-5} M) in acetonitrile upon irradiation with a white light LED through a 435LP filter (power = 15 mW cm^{-2}, λ_{exc} > 435 nm, irradiation from 0 to 3300 s).

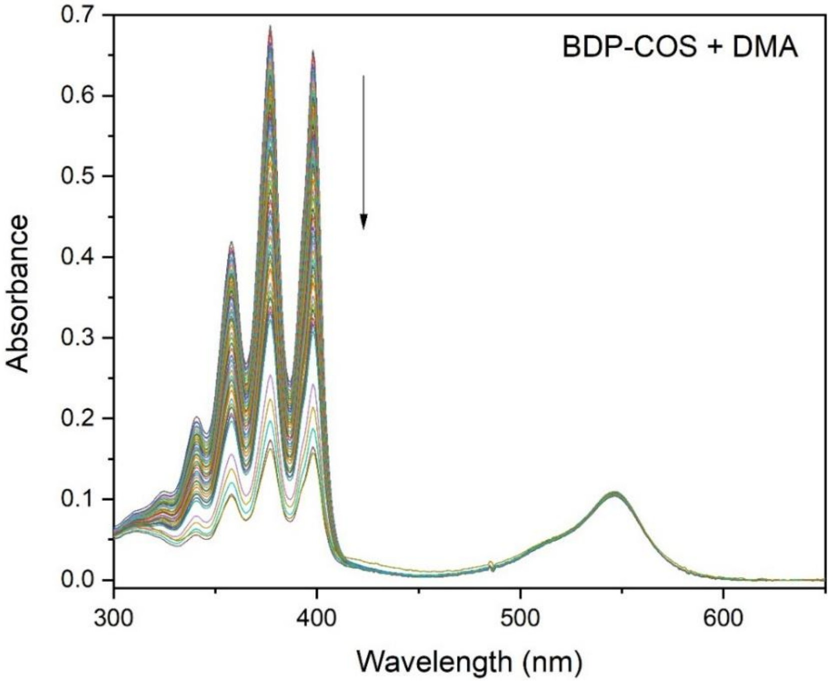

Figure S23. Variation of the absorption spectrum of a mixture of **BDP-COS** (c = 2.7 10^{-6} M) + DMA (c = 6.0 × 10^{-5} M) in acetonitrile upon irradiation with a white light LED through a 435LP filter (power = 15 mW cm^{-2}, λ_{exc} > 435 nm, irradiation from 0 to 3600 s).

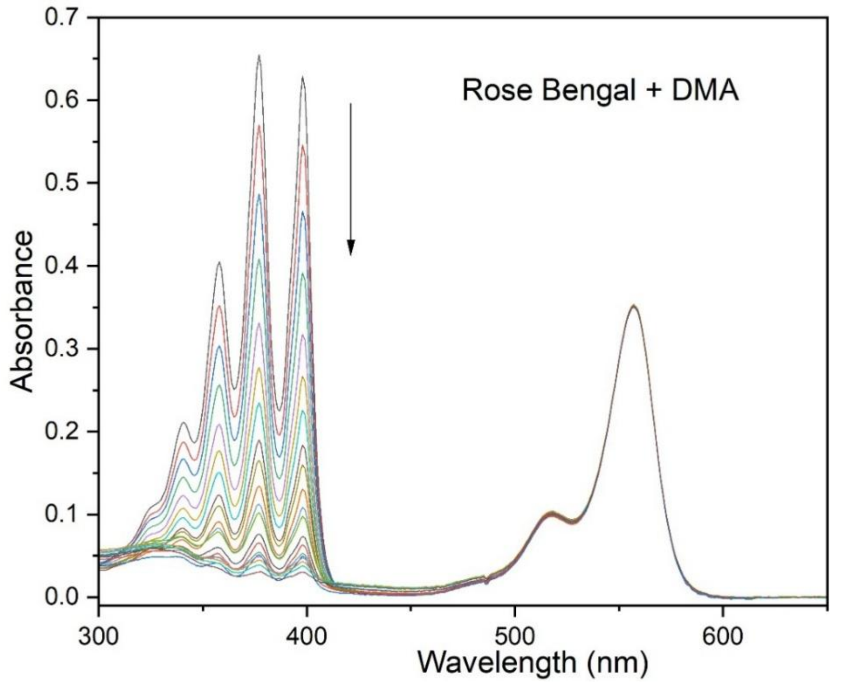

Figure S24. Variation of the absorption spectrum of a mixture of **Rose Bengal** (c = 2.7 10^{-6} M) + DMA (c = 6.0 × 10^{-5} M) in acetonitrile upon irradiation with a white light LED through a 435LP filter (power = 15 mW cm^{-2}, λ_{exc} > 435 nm, irradiation from 0 to 600 s).

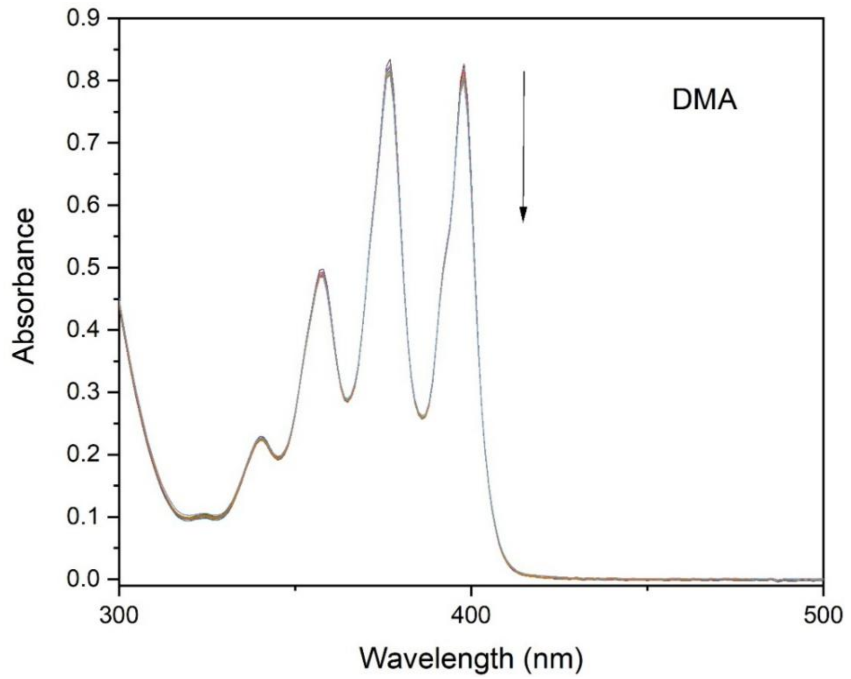

Figure S25. Variation of the absorption spectrum of DMA in acetonitrile upon irradiation with a white light LED through a 435LP filter (power = 15 mW cm^{-2}, λ_{exc} > 435 nm, irradiation from 0 to 600 s).

REFERENCES

[1] R. Núñez, M. Tarrés, A. Ferrer-Ugalde, F.F. De Biani, F. Teixidor, Electrochemistry and Photoluminescence of Icosahedral Carboranes, Boranes, Metallacarboranes, and Their Derivatives, Chem Rev 116 (2016) 14307–14378. https://doi.org/10.1021/acs.chemrev.6b00198.
